# Uncovering cancer vulnerabilities by machine learning prediction of synthetic lethality

**DOI:** 10.1186/s12943-021-01405-8

**Published:** 2021-08-28

**Authors:** Salvatore Benfatto, Özdemirhan Serçin, Francesca R. Dejure, Amir Abdollahi, Frank T. Zenke, Balca R. Mardin

**Affiliations:** 1 BioMed X Institute (GmbH), Im Neuenheimer Feld 583, 69120 Heidelberg, Germany; 2 Division of Molecular and Translational Radiation Oncology, National Centre for Tumour Diseases (NCT), Heidelberg University Hospital, 69120 Heidelberg, Germany; 3 Translational Innovation Platform Oncology & Immuno-Oncology, Merck KGaA, Frankfurter Str. 250, 64293 Darmstadt, Germany

## Abstract

**Background:**

Synthetic lethality describes a genetic interaction between two perturbations, leading to cell death, whereas neither event alone has a significant effect on cell viability. This concept can be exploited to specifically target tumor cells. CRISPR viability screens have been widely employed to identify cancer vulnerabilities. However, an approach to systematically infer genetic interactions from viability screens is missing.

**Methods:**

Here we describe PAn-canceR Inferred Synthetic lethalities (PARIS), a machine learning approach to identify cancer vulnerabilities. PARIS predicts synthetic lethal (SL) interactions by combining CRISPR viability screens with genomics and transcriptomics data across hundreds of cancer cell lines profiled within the Cancer Dependency Map.

**Results:**

Using PARIS, we predicted 15 high confidence SL interactions within 549 DNA damage repair (DDR) genes. We show experimental validation of an SL interaction between the tumor suppressor CDKN2A, thymidine phosphorylase (TYMP) and the thymidylate synthase (TYMS), which may allow stratifying patients for treatment with TYMS inhibitors. Using genome-wide mapping of SL interactions for DDR genes, we unraveled a dependency between the aldehyde dehydrogenase ALDH2 and the BRCA-interacting protein BRIP1. Our results suggest BRIP1 as a potential therapeutic target in ~ 30% of all tumors, which express low levels of ALDH2.

**Conclusions:**

PARIS is an unbiased, scalable and easy to adapt platform to identify SL interactions that should aid in improving cancer therapy with increased availability of cancer genomics data.

**Supplementary Information:**

The online version contains supplementary material available at 10.1186/s12943-021-01405-8.

## Introduction

Synthetic lethality occurs when simultaneous perturbation in two or more genes leads to cell death, whereas individual inactivation of single genes is still compatible with cell survival [1]. This phenomenon, first described in *Drosophila melanogaster* [2, 3], has been used as an approach in cancer therapy to exploit vulnerabilities of cancer cells by identifying druggable targets that when ablated or inhibited would selectively impact the viability of aberrant cancer cells [4]. The first successful therapy based on this approach is the use of Poly-ADP-ribose polymerase 1 (PARP1) inhibitors in patients deficient in homologous recombination pathway [5]. This discovery promoted the search for additional synthetic lethal (SL) targets in cancer research.

Studies in human cancer cell lines have accumulated multiple layers of genetic information that can be used to study SL interactions. This includes CRISPR/Cas9-based KO screens, RNAi and drug screens together with gene expression, mutation and copy number variation data. For instance, data from the Achilles project (part of the DepMap consortium [6]), based on both CRISPR-Cas9 and shRNA screens, have been exploited to uncover new potential SL interactions and several computational approaches have been developed for this purpose [7–13]. In addition, prioritization of cancer therapeutic targets has been proposed [14] based on a priority score derived from experimental evidence, the significance of fitness deficiency, target gene expression, target mutational status and evidence for other fitness genes in the same pathway. Based on these efforts, recently the Werner syndrome helicase (WRN) was found as a promising SL target in microsatellite instable cancers [14–17]. Despite the depth of information acquired on individual cancer cell lines only a few potential SL interactions have been identified so far. Due to their genomic complexity, it has been challenging to disentangle the genetic dependencies of tumor cell lines. Several of the approaches used to infer SL interactions are based on the assumptions of gene co-inactivation, mutual exclusivity and/or co-expression and are generally tested on mutational or gene expression data with traditional statistics (e.g. Wilcoxon rank sum test) or restricted to certain tissues [7, 8, 11].

Most of the current approaches for SL predictions are based on a massive number of multiple univariate statistical tests. This can lead to high number of false positives or after multiple-testing correction, to high number of false negatives. These models also do not address multicollinearity, a phenomenon in which the multiple predictors are highly correlated. For this reason, they may fail to predict potential feature associations by testing genetic interactions individually and they are not designed to find non-linear correlations. A major challenge is that the omics data are often composed of a relatively small number of samples and a high number of variables (often referred to as features or predictors). This type of data is also referred to as “big-p, little-n” (*p* >  > n), presents a problem of high dimensionality. A good strategy to tackle this problem is to reduce the number of dimensions through feature selection. By grouping similar features or filtering redundant ones, direct relationships between the response and the predictor can be readily identified. Feature selection methods can be grouped into (i) minimal-optimal methods that aim to find the minimal optimal subset of features to maximize the accuracy of the model, and (ii) all-relevant methods, that find all the relevant features to describe the outcome variable [18]. If the first ones often drop redundant (collinear) variables and suffer from inherent bias, the latter ones are able to capture all the important features to explain a phenomenon and have been successfully applied in bioinformatics, e.g. gene and single nucleotide polymorphism selection [19]. Random Forest (RF) algorithms are thus very suitable for *p* >  > n datasets, they require minimal tuning, no transformation of variables and they are robust to noise [20].

In particular, the robustness of the RF methods has been previously tested with gene expression datasets, whereby they showed high reliability in terms of post-selection classification accuracy and consistency.

Previous methods [7–13] provided very important steps towards the identification of targetable genetic interactions. For instance, Jerby-Arnon, L. et al. developed an approach (DAISY- data mining synthetic lethality identification pipeline) that led to the discovery of SL partners for the tumor suppressor *VHL*. Sinha, S. et al. presented MiSL (Mining Synthetic Lethals), an algorithm to identify mutation-specific SL partners in cancers and identified a SL interaction between the isocitrate dehydrogenase 1 (*IDH1)* mutation and the acetyl-CoA carboxylase 1 (*ACACA)*. Apaolaza, I. et al. employed the concept of genetic minimal cut sets and gene expression data to predict metabolic vulnerabilities in cancer and study the ribonucleotide reductase catalytic subunit M1 (*RRM1)* inhibition in myeloma cell lines. Lee, J. S. et al. developed an algorithm for identification of clinically relevant SL interactions (ISLE) using The Cancer Genome Atlas (TCGA) data. Despite these advances, an algorithm that can address the importance of each individual gene deficiency in explaining the dependencies observed in cancer cells in a tissue-independent manner is still missing. Here we present PAn-canceR Inferred Synthetic lethalities (PARIS), a machine learning approach that can predict potential vulnerabilities in cancer. The core of the workflow is a feature selection step, performed with RF algorithms that assign an importance score to each genomic feature based on the measured effect of knocking out a specific gene across hundreds of cancer cell lines. We initially established and tested PARIS to identify SL interactions among known DNA damage response (DDR) genes. We then expanded our search space and investigated the vulnerabilities of the DDR genes with the rest of the genome. We identified and confirmed two previously uncharacterized SL interactions. To allow straightforward exploration of the entire dataset, we generated an interactive web application. In summary, PARIS can be used as a platform to directly link certain genetic features to the viability data obtained after knocking out a specific gene and to uncover the meaningful relationships that represent potential cancer vulnerabilities.

## Results

### PARIS methodology

**Fig. 1:**
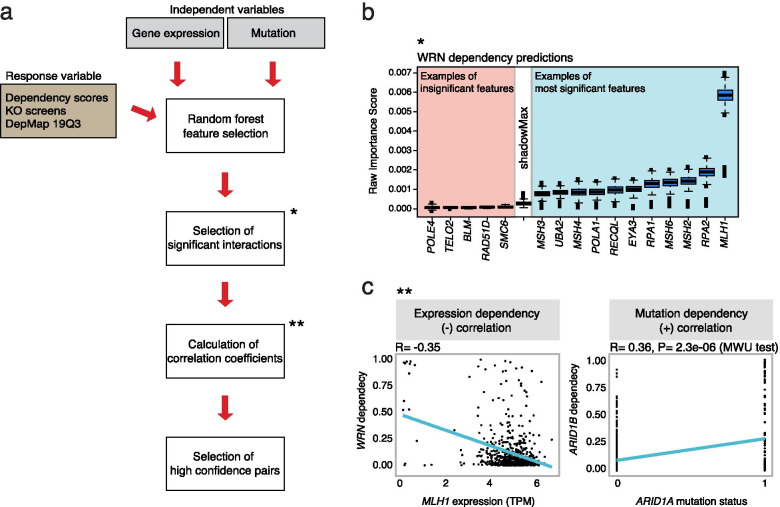
Workflow of PARIS (Pan cAnceR Inferred Synthetic lethalities). **a** The PARIS pipeline uses data retrieved from the DepMap consortium. Dependency scores from the CRISPR-Cas9 screens and mutation/expression data were used as response variables and as independent variables, respectively. Only damaging mutations, TCGA hotspots and predicted pathogenic (coding score from FATHMM > 0.7) mutations were considered. The RF feature selection step assigns important scores to each feature (mutations and expression independently) to describe the dependency scores of a particular gene. The significant-selected pairs are optionally filtered based on the direction of the relationship: positive for mutations/dependencies and negative for expression/dependencies. Candidates for SL gene pairs are ranked based on their importance scores. **b** The RF feature selection is based on the Boruta algorithm, which selects significant features with importance scores higher than the maximum importance score obtained by random probes during the iteration process (shadowMax). In the example *WRN* dependency is explained by multiple genes belonging to the mismatch repair pathway that have significantly higher importance scores than the random probes. **c** Examples of dependency/selected features correlations. Scatterplots show the negative correlation between *WRN* dependency/*MLH1* expression and the positive correlation between *ARID1B* dependency/*ARID1A* mutation status

We developed PARIS as a computational approach to infer vulnerabilities in cancer cells. The core of the pipeline is based on the RF algorithms to assess importance of each independent variables (mutation or gene expression) with respect to the response variable (dependency scores). As the response variable, we retrieved gene dependency score data based on the CERES pipeline that can estimate the gene essentiality levels from CRISPR-Cas9 screen results correcting for the gRNA activity and the copy-number effect [21]. Additionally, we retrieved the mutation and expression data from the Cancer Cell Line Encyclopedia (CCLE), part of the DepMap consortium. In order to filter for relevant mutations, we only considered pathogenic mutations based on the FATHMM (Functional Analysis through Hidden Markov Models) predictions [22], hotspot mutations reported in TCGA or mutations that are already annotated as damaging. Using these data, we applied a feature selection step, based on a machine learning algorithm that aims to explain certain gene dependencies from the CRISPR-Cas9 screens by mutation or misregulation of genes across hundreds of cancer cell lines (Fig. 1a). For this purpose, we selected the Boruta algorithm [23] due to its robustness and adaptability to omics data [24]. This algorithm uses the RF importance scores and iteratively removes any features that are found to be significantly less relevant (i.e. show a lower importance score) than random probes (Fig. 1b).

To benchmark PARIS, we initially compared two importance measures to select significant features:

i) the mean decrease of impurity (Gini importance), obtained summing the total nodes impurity reduction where the variable appears, and ii) the mean decrease of accuracy (raw permutation importance), measuring accuracy reduction on out-of-bags samples when the values of the variable are randomly permuted.

### Quality assessment of the importance scores

As a proof of concept, we initially focused on known DDR-related genes due to their relevance as potential targets in cancer treatment. We manually curated a list of 549 genes [25, 26] for which we used the dependency scores across 625 cancer cell lines as response variables, as well as the mutational and expression data as features/predictors. We then selected pathogenically mutated or misregulated genes that can explain the dependencies of each genetic target in the CRISPR-Cas9 screens using the Boruta algorithm. We extracted the selected pairs of genes along with the importance scores retrieved from the feature-selection step (Fig. 1b). For instance, we found that *MLH1* was one of the most significant features to explain *WRN* dependency, as previously described [14–17]. In order to identify interactions that could represent potential SL interactions or vulnerabilities in cancer cell lines, we focused on positive relationships in the case of dependency/mutation pairs and on negative relationships for dependency/expression pairs, using the Pearson correlation coefficient (PCC) score to retrieve the direction of the relationship. We used PCC due to its well suitability for data with high class imbalances such as the gene dependencies in cancer cells, whereby the dependent group is much smaller than the non-dependent group. For example, the *WRN* dependency scores negatively correlated with *MLH1* expression and *ARID1B* dependency score positively correlated with *ARID1A* mutation status, as expected from previous reports [27, 28], demonstrating the capability of PARIS to detect SL interactions (Fig. 1c). We selected all the significant importance scores and subsequently, we scaled the importance scores between 0 and 1 within each group, where the most significant interaction was scaled to 1. We next investigated the concordance between the importance scores derived from different algorithms.

**Fig. 2:**
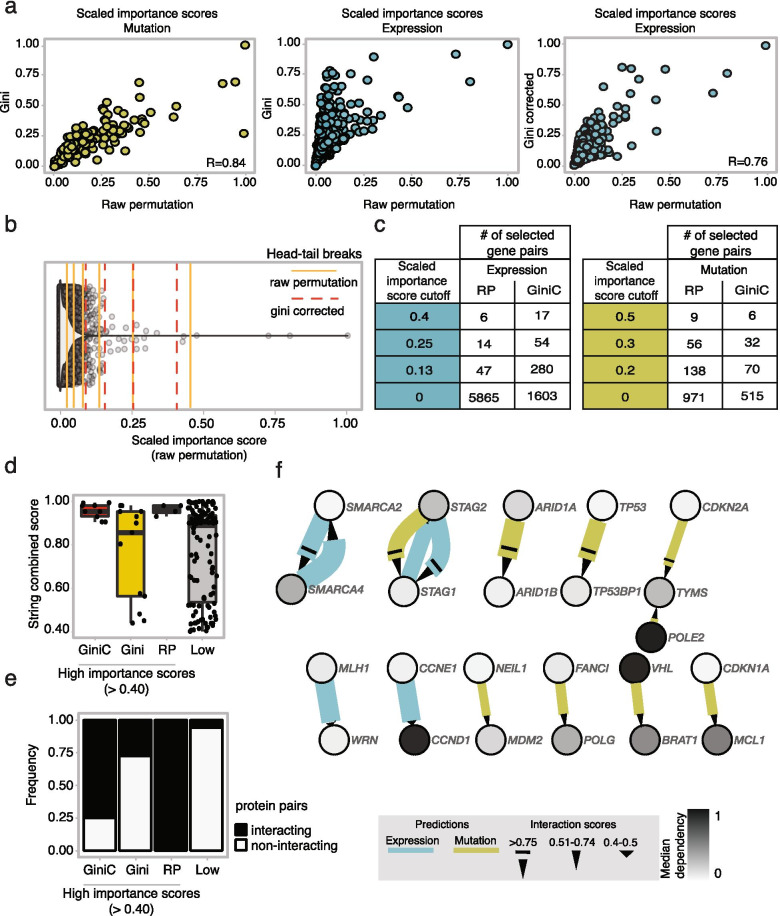
Importance score quality assessment and potential synthetic lethal interactions among DDR genes. **a** Comparisons of importance score calculation methods on commonly selected DDR gene pairs. Gini—raw permutation scaled importance scores correlation using mutation features (green) and Gini/Gini corrected—raw permutation scaled importance scores correlation with expression features (blue). **b** Density distribution of the raw permutation scaled importance scores with superimposed breaks obtained by the Head/Tail breaks algorithm using raw permutation (yellow lines) and Gini corrected (dotted red lines) importance score methods. **c** Number of selected gene pairs above different scaled importance score cutoffs based on expression (blue) or mutation (green) features. **d** STRINGdb combined scores of interacting gene pairs selected with high confidence (scaled importance score > 0.4) by the three approaches and with low confidence (scaled importance score < 0.4) by all of the methods. **e** Percentage of interacting gene pairs over the selected ones in the four described groups. **f** Network of predicted SLs among DDR genes based on the raw permutation importance score. Each node represents a gene and each edge a relationship; the arrow starts from the mutated (green) or dysregulated gene (blue) and arrives to the gene showing an associated increased dependency score. The width is proportional to the absolute value of the Pearson correlation coefficient. The color of the node shows the median of the dependency score of the gene in a grey scale. Different arrow shapes show three levels of confidence scores based on the scaled importance scores

Considering only the interactions selected by both algorithms, the two scaled importance scores showed a strong correlation (R = 0.84, PCC) for predictions with mutations, however a moderate (R = 0.58, PCC) correlation for the expression-based predictions (Fig. 2a). We reasoned that these differences may stem from the fact that the expression data allow many more splitting points during the tree construction process implemented by PARIS compared to the mutation-based data. Since the Gini metrics can be prone to assign higher scores on predictions based on the expression data, we hypothesized that it may introduce biases. For this reason, we applied an additional RF algorithm using a corrected impurity (corrected Gini) importance score [29] to improve the feature selection step (Fig. 2a). This metric is able to reduce biases in the measurement and is suggested to be as accurate as the raw permutation one with the advantage of reduced computational time (see Methods).

The scaled importance scores of the selected pairs of genes from the RF feature selection step showed long-tailed distributions in all the groups (Supplementary Fig. 1a) indicating that most of the interactions were inferred with low importance scores and fewer relationships were assigned high scores. To select gene pairs with the highest confidence, we defined classes of importance scores based on the head/tail breaks clustering method, which is a clustering algorithm well suited for data with heavy-tailed distributions (see Methods, Fig. 2b and Supplementary Fig. 1a).

For example, using the raw permutation method in the expression dataset, out of 5865 gene pairs that passed the *P* value threshold (*P* < 0.01), 14 gene pairs had scaled importance score of more than 0.25. On the other hand, the corrected Gini method identified less gene pairs that passed the *P* value cutoff, however more gene pairs were identified with higher importance scores (Fig. 2c).

Based on these analyses, we considered the last break point of the head/tail breaks clustering method as the threshold for identifying the gene pairs with the highest confidence. We then investigated the robustness of the raw permutation, Gini and corrected Gini methods to identify interacting genes with high confidence (scaled importance score more than 0.4). For this we retrieved the interaction information from the STRING database [30], whenever available, as a proxy for the strength of the genetic interactions. In addition, we considered a fourth group including all the pairs selected with low confidence (scaled importance score less than 0.4) by all of the three approaches (Supplementary Fig. 1b). We first investigated the gene pairs identified from the expression-based data and compared the combined scores from the STRING database indicating the likelihood of protein–protein interactions. We observed higher combined scores in the groups belonging to the corrected Gini and the raw permutation scores than to the Gini and the “low confidence” groups (Fig. 2d). When we considered only the experiment-based interaction score, as the most stringent source of evidence, the raw permutation high confidence-selected pairs showed highest scores. However, in general, the percentage of interacting protein pairs was much higher in both the corrected Gini and raw permutation groups (Fig. 2e). As expected, we did not observe any differences among the three “high confidence” groups when we used the mutation data. Nonetheless, only the “low confidence” group showed lower values in terms of combined scores, experiment-based interaction score and percentage of interactions (Supplementary Fig. 2a, b). Based on these observations, we concluded that the raw permutation method is very robust in identifying gene pairs with high confidence and the corrected Gini method can drastically improve the confidence of selection, in particular when the expression data are used as independent variables. In addition, we demonstrated that the scaled importance scores can be an effective measure for the confidence of the predicted SL interactions by PARIS.

### Prediction of synthetic lethal interactions among DDR genes

For intuitive data browsing and visualization we built an R shiny [31] app based on PARIS results. In this app, the selected gene pairs are represented as a directed graph, in which the arrows point to the dependent genes of the pairs starting from the deficient ones (mutated or dysregulated). Users can apply different filters, e.g. to the scaled importance scores and type of features to explore the interactions and export the results as data tables (see Methods for details and Additional file 1 for the complete table). In the context of the DDR genes, to select and investigate the pairs inferred with highest confidence, we used scaled importance score thresholds of 0.4 and 0.5 for expression and mutation-based predictions, respectively. Several of the gene pairs identified from this cohort are paralogs and function in protein complexes that are intrinsically related and more likely to show SL interactions (Supplementary Fig. 2c). As previously suggested, the buffering relationships between paralog pairs can explain a significant higher probability to display synthetic lethality, in agreement with our results in which the top high score pair group mostly consists of paralog pairs [32]. In addition, among others, notable examples of high confidence SL predictions based on mutation data were *ARID1B*-*ARID1A*, *STAG1*-*STAG2* and *TYMS*-*CDK2NA*. When considering the expression data, we again observed a high confidence interaction between *STAG1*-*STAG2* in addition to a bidirectional interaction between *SMARCA2* and *SMARCA4* (Fig. 2f, Supplementary Fig. 3a, b). Interestingly, the combination of deficiencies of the genes *ARID1B*-*ARID1A* and *SMARCA2*-*SMARC4*/ *SMARCA4*-*SMARCA2*, all belonging to the SWI/SNF chromatin remodeling complex, has already been demonstrated to show SL interactions [27, 28, 33–35]. Additionally, the SL interaction between *STAG1*-*STAG2* has previously been described [36].

### PARIS predicts TYMS dependency of cancer cell lines

**Fig. 3:**
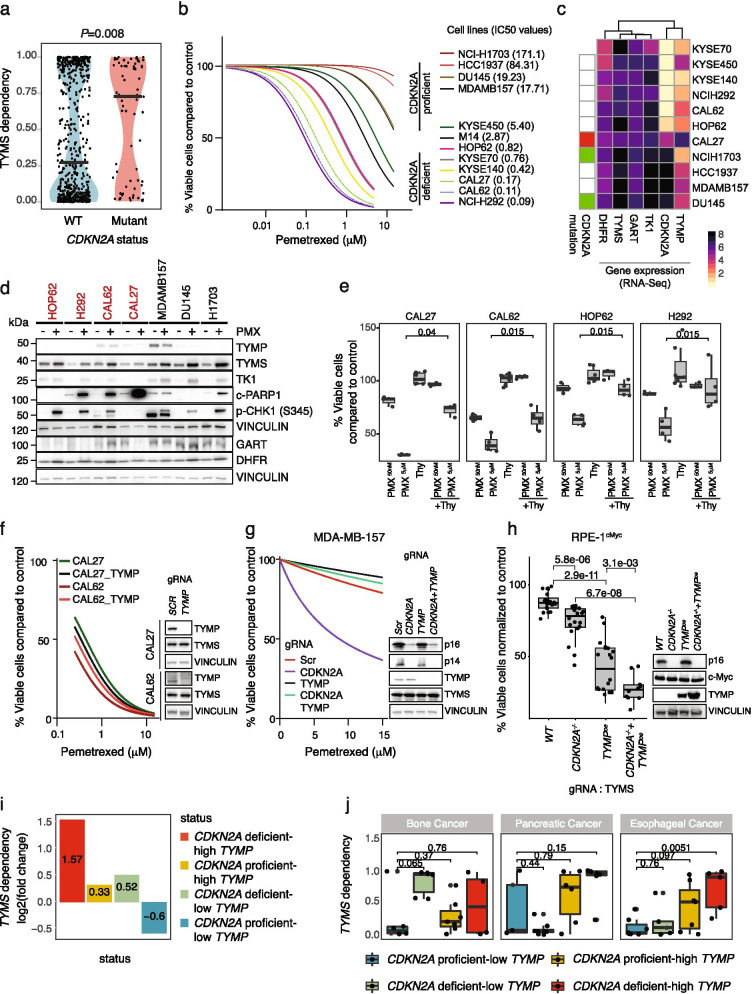
Synthetic Lethal interaction between *CDKN2A* and *TYMP* with *TYMS.***a** Violin plot showing *TYMS* dependency (0 lowest, 1 highest) with respect to mutation status of CDKN2A in DepMap data. Each point represents corresponding cell line and dependency value. *P*-value was calculated using Mann–Whitney U test. **b** A panel of cancer cell lines carrying wildtype *CDKN2A*: MDA-MB-157, HCC1937, missense mutant: DU-145, NCI-H1703, nonsense mutant: CAL27, deleted for *CDKN2A* locus: CAL62, HOP62, NCI-H292, KYSE-140, KYSE-70, KYSE-450, splice site mutant: M14, were tested with increasing concentrations of PMX (0, 0.001, 0.01, 0.05, 0.1, 0.25, 0.5, 1, 5 and 15 μM). Cell viability was measured with CellTiter Glo after incubation of the cells with PMX for 96 h. Drug response curves were generated and IC50 values shown in brackets (μM) next to each cell line were calculated from at least 3 biological and 9 technical repeats. **c** Heatmap showing CDKN2A mutation status (red box = nonsense mutation, green boxes = missense mutation, white boxes = WT); *CDKN2A, TYMP, TYMS, GART, DHFR* expression status for cell lines used in this study. Color scale corresponds to (log2(TPM) + 1) values based on RNA-Seq. **d** Cancer cell lines were treated with PBS or 5 μM PMX for 48 h. Western blot was performed for the proteins involved in Thymidine nucleotide metabolism (DHFR, GART, TYMS, TK1 and TYMP), DNA damage checkpoint marker (phospho-CHK1 (S345)) and apoptosis marker (cleaved-PARP1). VINCULIN served as a loading control. Cell lines labeled with red color are *CDKN2A*-deficient and show sensitivity to PMX in (b). Quantification of these blots are available in Additional file 2. **e** PMX-sensitive cancer cell lines were supplemented with PBS or 50 μM of thymidine to the media during PMX (50 nM or 5 μM) treatment. Cell viability was measured using live-cell protease (CellTiter Fluor) and % viability was calculated compared to the control treatment. Boxplots were generated from data from at least 3 biological and technical repeats. In the boxplots, centerlines mark the medians, box limits indicate the 25^th^ and 75^th^ percentiles, and whiskers extend to 5^th^ and 95^th^ percentiles. *P*-values were calculated using Mann–Whitney U test. **f** CAL27 and CAL62 cells were transfected with gRNA targeting *TYMP*. Five days post transfection, control or *TYMP* KO cells were treated with increasing doses of PMX (0, 0,25, 0.5, 1, 5 and 15 μM) for 96 h. Drug response curves were generated using data from 8 and 2 biological replicates, respectively. (Right) Western blot analysis of the indicated proteins 5 days post gRNA transfection. **g** MDA-MB-157 cells were transfected with gRNA targeting *TYMP*, *CDKN2A* or both genes. Five days post transfection, control or KO cells were treated as described in (f). Drug response curves were generated from at least 3 biological replicates. (Right) Western blot analysis of the indicated proteins 5 days post gRNA transfection. **h** RPE1 ^*TP53−/−; CMYC*^, RPE1 ^*TP53−/−; CMYC;CDKN2A−/−*^, RPE1 ^*TP53−/−; CMYC; TYMP*^, RPE1 ^*TP53−/−; CMYC; TYMP;CDKN2A−/−*^,cells were transfected with two different gRNA against *TYMS* and viability were measured 7 days using CellTiter Glo. Values were normalized to scrambled gRNA transfection and were plotted from at least 9 biological replicates. In the boxplots, centerlines mark the medians, box limits indicate the 25^th^ and 75^th^ percentiles, and whiskers extend to 5^th^ and 95^th^ percentiles. *P*-values were calculated using a Mann–Whitney U test. (Right) Western blot analysis of the generated RPE-1 cell lines. **i** Prediction of *TYMS* dependency by different genetic backgrounds. DepMap cancer cell lines grouped by their *CDKN2A* mutation and *TYMP* expression status. In each group the ratios of the percentage of *TYMS* dependent/*TYMS* independent cell lines were calculated and plotted. **j** *TYMS* dependency distribution is shown as boxplots. Cell lines are grouped by their *CDKN2A* and *TYMP* expression status. *CDKN2A* deficiency/proficiency is defined by the presence of a mutation or copy number loss and *TYMP* status is defined by tissue as high and low expressed using the median as a cut-off

Having established that PARIS can identify high confidence SL interactions, we next focused on the previously uncharacterized *TYMS*-*CDKN2A* vulnerability identified in our analysis. PARIS predicted cells with *CDKN2A* damaging mutations to show sensitivity to *TYMS* knockout (Fig. 2f, and Fig. 3a). The cyclin dependent kinase inhibitor 2A (*CDKN2A*) is a ubiquitously expressed tumor suppressor gene. It encodes two proteins, p14^Arf^ and p16^INKA^, also referred to as p14 and p16, respectively. Both proteins act as tumor suppressors and are involved in cell cycle regulation. In the case of inactivating germline mutations, *CDKN2A* is one of the DDR-related genes associated with inherited/familial predisposition for melanoma, glioblastoma multiforme and pancreatic cancer. In addition, *CDKN2A* is also frequently somatically mutated in various cancers [25]. The roles of CDKN2A in cell cycle pathways make it crucial for maintaining genomic stability and as a result, aberrations of it leads to defects in cell cycle regulation and senescence, contributing to tumorigenesis and poor disease prognosis [37]. Thymidylate Synthase (TYMS) is the enzyme that converts deoxyuridine monophosphate (dUMP) into deoxythymidine monophosphate (dTMP). This step is followed by the phosphorylation of dTMP to dTTP, which is in turn incorporated into DNA during DNA synthesis. TYMS plays an important role in replenishing the nucleotide pool for replication. Due to its pivotal role in the de novo synthesis of pyrimidines, TYMS is an established drug target for cancer treatment [38]. For instance, a new generation of antifolates targeting TYMS such as Pemetrexed (PMX) or Raltitrexed (RTX) are used for the treatment of squamous cell carcinomas of the lung and mesotheliomas among many other tumors [39, 40]. In addition, overexpression of *TYMS* is associated with resistance to PMX. However, how *TYMS* levels are regulated in cancer is not yet well understood [41, 42].

To confirm the PARIS predictions and test the sensitivity of cancer cells to TYMS inhibition, we treated a panel of cell lines with different *CDKN2A* genetic backgrounds with increasing doses of PMX (ranging from 10 nM to 15 μM) and measured cell viability as a readout for synthetic lethality. Even though the PARIS predictions were based on pathogenic mutations of *CDKN2A*, we observed pronounced PMX sensitivity not only in cell lines that carry a nonsense mutation in the *CDKN2A* gene (e.g. CAL27) but also in cell lines that have a homozygous *CDKN2A* deletion (e.g. H292), whereas this effect was not observed in any *CDKN2A*-proficient cell lines (Fig. 3b,c). Importantly, the IC50 values derived from these assays varied significantly between the *CDKN2A* proficient and deficient cells with a median IC50 of 51.77 μM for *CDKN2A*-proficient cells and 0.59 μM for *CDKN2A*-deficient cells (*P* = 0.004, Mann–Whitney U test). To better understand the response of *CDKN2A* mutant or deleted cancer cell lines to PMX we next performed immunoblots of the proteins involved in the thymidine nucleotide metabolism pathway, including TYMS, Thymidine phosphorylase (TYMP), thymidine kinase 1 (TK1), glycinamide ribonucleotide formyltransferase (GART) and dihydrofolate reductase (DHFR) as well as cleaved PARP1, and p-CHK1 as markers of apoptosis and DNA damage checkpoint activation, respectively. Upon PMX treatment, irrespective of their *CDKN2A* status, we observed that cells upregulated TYMS, TK1 and DHFR protein levels at least by twofold (Fig. 3d and Additional file 2). *TYMS*, *TK1* and *DHFR* levels are known to be regulated during cell cycle [43–45] and the increase in their protein levels is possibly due to accumulation of cells in S phase upon PMX, since we observed increased nuclear CyclinA levels following PMX treatment (Supplementary Fig. 4a). Interestingly, we observed higher baseline GART levels in *CDKN2A*- proficient cells (Fig. 3d, Additional file 2). Consistent with the viability results, *CDKN2A*-deficient cells that are most affected by PMX treatment such as CAL27, CAL62 and NCI-H292, strongly induced apoptosis upon PMX treatment (Fig. 3d). Importantly, sensitivity to either low (50 nM) or high doses (5 μM) of PMX in these cells was rescued by thymidine supplementation, as previously suggested [39] (Fig. 3e, Supplementary Fig. 4b). Thymidine can be converted into dTMP by both the cytosolic and mitochondrial enzymes thymidine kinases (TK1 and TK2, respectively) [46], thus strongly suggesting that the cell death occurs due to the depletion of dTMP through the predicted SL interaction.

Notably, TYMP overexpression was observed in some of the cell lines that show increased sensitivity to PMX, such as CAL27 and CAL62, which carry either a nonsense mutation in the *CDKN2A* gene or a deletion of the *CDKN2A* locus, respectively (Fig. 3c). It has been reported that cells overexpressing *TYMP* are more sensitive to *TYMS* depletion [47]. TYMP functions within the pyrimidine salvage pathway by converting thymidine into thymine, thus it plays a key role in regulating thymidine levels and dTMP production [48]. Since PARIS also predicted that *TYMP* overexpression is linked to *TYMS* dependency (Supplementary Fig. 4c-f), we tested the direct involvement of *TYMP* overexpression in PMX sensitivity in the CAL27 and CAL62 cell lines that are *CDKN2A*-deficient. Transfection of gRNAs targeting *TYMP* in CAL27 or CAL62 cells stably expressing Cas9 did not lead to any changes in PMX sensitivity as compared to the control (scrambled) gRNA transfected cells, suggesting that *TYMP* expression alone is not sufficient to control *TYMS* dependency (Fig. 3f). To delineate the involvement of TYMP and p14/p16 in regulating PMX sensitivity, we used the MDA-MB-157 cell line, in which we observed sufficiently high levels of p14/p16 and TYMP and transfected gRNAs targeting *TYMP* and *CDKN2A*. While depletion of *TYMP* showed no additional sensitization to PMX, depletion of *CDKN2A* dramatically increased the sensitivity of these cells to PMX. This could be due to misincorporation of deoxyuridine during DNA synthesis and apoptosis upon PMX treatment or replication stress due to dTTP depletion as indicated by increased cleavage of PARP-1 and accumulation of cells in S phase (Fig. 3g, Supplementary Fig. 4 g, h). Strikingly, knockout (KO) of *TYMP* in *CDKN2A*-deleted MDA-MB-157 cells rescued their sensitivity to PMX and decreased apoptosis, suggesting a genetic interaction between *TYMP* and *CDKN2A* (Fig. 3g, Supplementary Fig. 4 g).

While PMX’s known primary target is TYMS, at higher concentrations it is also known to inhibit additional enzymes such as DHFR and to a lesser extent GART. To study the relative contributions of *TYMP* and *CDKN2A* without PMX in a stable genetic background, we established a model cell line that resembles tumorigenic growth based on retinal pigment epithelial-1 (RPE-1) cells by stable overexpression of *c-myc* and generated isogenic cell lines with *CDKN2A* deletion and/or *TYMP* overexpression. In these cell lines we then tested the effects of *TYMS* knockout on cell viability. In *CDKN2A* KO cells gRNAs targeting TYMS led to a mild but significant decrease in cell viability. The negative effect on cell viability was also observed by *TYMP* overexpression and was significantly exacerbated by combined overexpression of TYMP and CDKN2A deficiency (Fig. 3h, Supplementary Fig. 4i). Collectively, these results point to an effect of *CDKN2A* depletion on PMX sensitivity together with *TYMP*. Indeed, categorizing DepMap data into 4 groups based on the *CDKN2A* status and *TYMP* gene expression levels, combination of *CDKN2A* deficiency and high *TYMP* expression best explains the TYMS sensitivity (Fig. 3i). However, it is possible that in different cell types, relative contributions of *TYMP* and *CDKN2A* to PMX sensitivity vary. Consistent with this idea, upon grouping the DepMap cancer cell lines according to tissue origin, we noted that CDKN2A status and TYMP expression have varying effects on controlling *TYMS* dependency in different tumor types. For instance, in bone cancer cell lines, deficiency of *CDKN2A* alone can explain the dependency to *TYMS*. On the other hand, dependency to *TYMS* in *CDKN2A*-mutant pancreatic cancer lines correlates with *TYMP* expression levels, while in esophageal cancer cell lines both *TYMP* expression levels and *CDKN2A* status together can better explain *TYMS* sensitivity (Fig. 3j, Supplementary Fig. 5 and 6). A notable exception was observed in lung tumor cell lines in which neither *TYMP* expression nor *CDKN2A* status affected the dependence of the cells to *TYMS* depletion (Supplementary Fig. 5 and 6). In summary, we propose that cells with *CDKN2A* deficiency and *TYMP* overexpression together contribute to *TYMS* sensitivity and that these dependencies can be cell-type specific.

### Prediction of vulnerabilities of DDR genes across the genome by PARIS

**Fig. 4:**
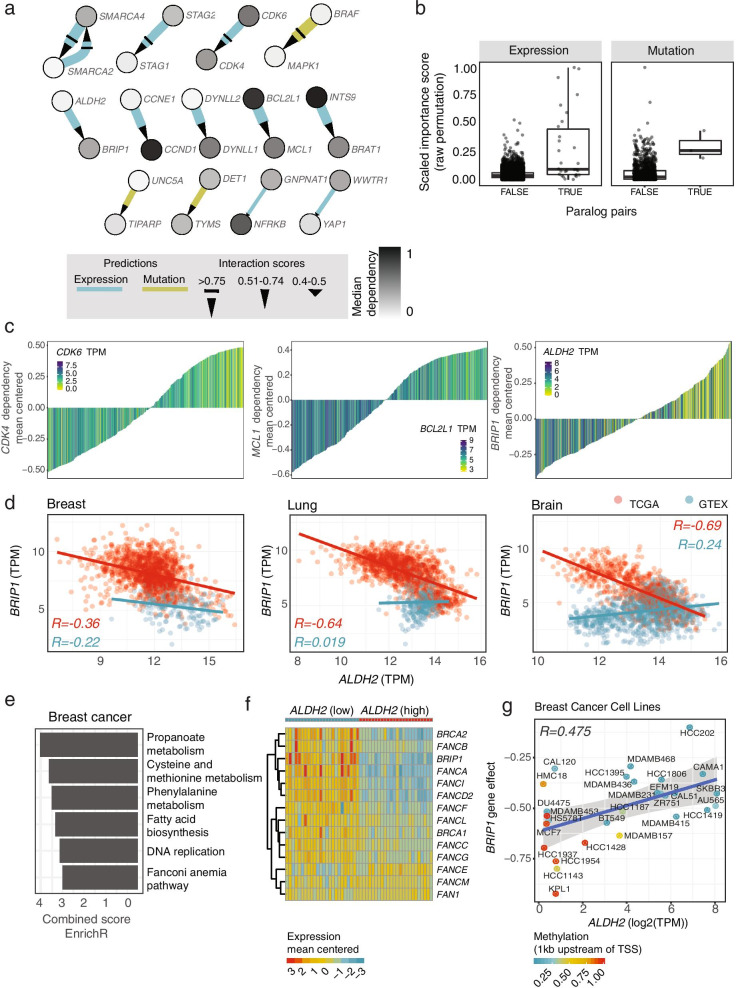
Vulnerabilities of DDR related genes. **a** Network of predicted SLs between DDR genes and the genome based on the raw permutation importance score. Each node represents a gene and each edge a relationship; the arrow starts from the mutated (green) or dysregulated gene (blue) and arrives to the dependent gene. The width is proportional to the absolute value of the Pearson correlation coefficient. The color of the node shows the median of the dependency score of the gene in a grey scale. Different arrow shapes show three levels of confidence based on the scaled importance score. **b** Scaled raw permutation importance score distributions of selected gene pairs divided into paralogs or not in the two cohorts (expression and mutation). **c** Bar plots showing examples of high-confidence predicted SL gene pairs using expression features. The ranked bars show the dependency scores (mean centered) of one gene across the cancer cell lines and the color gradient shows the expression level of the second gene. **d** Scatterplots showing the gene expression levels (based on RNA-Seq) of *BRIP1* and *ALDH2* in matched tumor (TCGA) and normal (GTEX) breast, lung and brain tissues. **e** Top altered pathways in the enrichment analysis of differentially expressed genes in TCGA cc samples expressing high or low *ALDH2*. **f** Heatmap showing expression levels (mean centered) of the main Fanconi anemia genes in TCGA breast cancer samples expressing high or low *ALDH2*. **g** Scatterplots indicating the correlation between the expression levels of *ALDH2* and *BRIP1* gene effect together with the promoter methylation levels of *ALDH2* in breast cancer cell lines

To demonstrate the ability of PARIS to select SL interactions in larger cohorts and to reveal interesting potential vulnerabilities among DDR and other genes, we extended the features of the dataset (both for mutation and expression) to all the available genes (~ 18,000) (Additional file 3). In this case, the Boruta algorithm with the raw permutation score was used to select the high-confidence pairs due to its reliability, as described above. Overall, the importance scores derived from the two cohorts’ predictions were consistent (Supplementary Fig. 7). For instance, *ARID1B*-*ARID1A*, *SMARCA2*-*SMARCA4* and *STAG1*-*STAG2* were also found as high-confidence predicted SLs in this larger cohort (Fig. 4a). Moreover, we identified well-known vulnerabilities in cancer, such as *MAPK1* dependency in *BRAF* mutated cells [49]. In agreement with the analysis performed within the DDR cohort, several of the predicted SL interactions are paralog genes (Fig. 4b), for instance *CDK4*-*CDK6* [50]. Besides these paralog pairs, PARIS also predicted with high-confidence recently identified SL interactions such as the one between the anti-apoptotic genes *MCL1* and *BCL2L1* [51–54] (Fig. 4c). In general, and consistent with earlier results, we predicted more SL interactions based on the expression data, suggesting that expression is a better predictor to explain the dependency scores from the CRISPR-Cas9 screens [55].

Among these high-confidence SL pairs, we identified a potential new vulnerability between *ALDH2* and *BRIP1* (Fig. 4c). The aldehyde dehydrogenase 2 (*ALDH2*) gene encodes a mitochondrial enzyme mainly involved in the detoxification of acetaldehyde [56]. BRCA1 interacting protein C-terminal helicase 1 (*BRIP1*) encodes a DNA helicase, also known as Fanconi Anemia group J protein (FANCJ), which plays a role within the Fanconi Anemia (FA) DNA repair pathway and has overall a broader function in maintaining genome stability by resolving DNA secondary structures [57]. The FA pathway is responsible for repairing interstrand crosslink (ICL) DNA damage, a type of lesion represented by a covalent bond between two complementary DNA strands. Such crosslinks cause genomic instability by interfering with the function of the replication machinery [58]. Acetaldehyde (ACE) is a highly reactive molecule produced from either exogenous sources, such as alcohol, cigarette smoke, environmental pollutants, or endogenous ones, such as intracellular metabolic reaction [59] and it represents a potential source of ICL formation [60, 61].

It has been shown that in hematopoietic stem cells, ALDH2 and the FA pathway provide two layers of protection against ACE-induced DNA damage [62–65], demonstrating an interesting link between the two pathways in the stem cell pool. However, the interplay between ALDH2 and BRIP1 or other components of the FA pathway in human tumors is less investigated. Based on the PARIS prediction, cancer cell lines that express low levels of *ALDH2* become dependent on *BRIP1*, possibly to balance a harmful increase in genomic instability. To understand how *ALDH2* and *BRIP1* levels are regulated in human tumors, we looked at cancer gene expression data obtained from the TCGA and compared them to those found in the normal tissue controls derived from the Genotype-Tissue Expression (GTEx) database. We found *ALDH2* to be downregulated in several tumor types. Moreover, we observed, particularly in breast, brain and lung cancer cohorts, a strong negative correlation between *ALDH2* and *BRIP1* (Fig. 4d). These data suggested a potential upregulation of *BRIP1* in *ALDH2*-low expressing tumor samples. Indeed, when we divided tumor samples based on their *ALDH2* expression levels and performed differential gene expression analysis, the FA pathway was one of the highly upregulated pathways among these two groups (Fig. 4e, Supplementary Fig. 8a, b), and the FA pathway components *BRIP1*, *FANCD2* and *FANCI* were consistently upregulated across different tumor types (Fig. 4f, Supplementary Fig. 8c, d) and independently of tumor stage (Supplementary Fig. 8e). These results suggest that the *ALDH2*-*BRIP1* vulnerability is important for a variety of tissue-specific cancers.

**Fig. 5:**
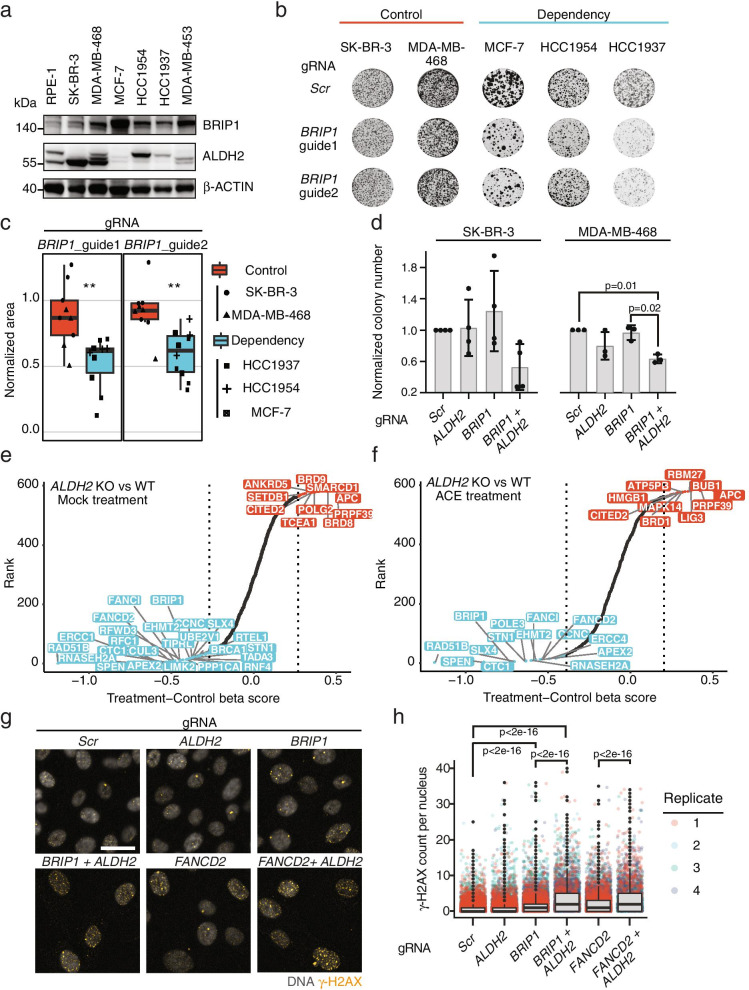
Validation of the dependency of low-*ALDH2* expressing cells on *BRIP1* expression. **a** Western blot of BRIP1 and ALDH2 in the indicated breast cancer cell lines. The lower migrating band corresponds to ALDH2. RPE-1 = RPE-1^*TP53*−/−^ hTERT cells. Data are representative of 3 independent experiments. **b** Colony formation assay images of the indicated cell lines stably expressing Cas9 and transfected with a scrambled gRNA or 2 independent gRNAs targeting *BRIP1*. Colonies were stained with crystal violet 15 days post-transfection. Images are representative of ≥ 3 independent experiments. **c** Colony formation assay quantification in the control or dependency cell lines. The scrambled KO colony area is used for normalization. In the boxplots, centerlines mark the medians, box limits indicate the 25^th^ and 75^th^ percentiles, and whiskers extend to 5^th^ and 95^th^ percentiles. *P*-values are calculated based on Mann–Whitney U test (** *p*  ≤ 0.01). **d** Colony formation assay quantification in MDA-MB-468 and SK-BR-3 stably expressing Cas9 and transfected with the indicated gRNAs. Bars represent normalized mean + standard deviation of 4 (SK-BR-3) or 3 (MDA-MB-468) independent experiments. The scrambled KO colony number is used for normalization. *P*-values are calculated with a one-way ANOVA test. Significant *p*-values are indicated. **e**, **f** Pooled CRISPR screen results. The beta scores differences between RPE^*TP53*−/−,ALDH2−/−^ and RPE^*TP53*−/−^ are showed as ranked plots, both in mock ( **e**) and ACE ( **f**) treatments conditions. Gene names with a negative beta score difference 2.5 bigger than the standard deviation are showed in light blue, top 10 gene names with a positive beta score difference are shown in light red. **g** Representative immunofluorescence images of RPE-1^*TP53−/−*^ cells stably expressing Cas9 and transfected with the indicated gRNAs. Cell images were acquired 6 days post-transfection. Nuclei are pseudocolored in gray; the yellow dots mark γ-H2AX foci. Scale bar = 20 μm. **h** Quantification of γ-H2AX foci formation in RPE-1^*TP53−/−*^ under the indicated conditions. Each dot indicates the number of foci/nucleus in each of the 4 biological replicates. *P*-values are calculated using a one-way ANOVA test. Selected significant *p*-values are indicated. The complete *p*-value list in provided in Additional file 4

To further validate this prediction, we selected a panel of breast cancer cell lines to test the vulnerability of low *ALDH2* expressing cells. Interestingly, several of the breast cancer cell lines showed promoter hypermethylation of *ALDH2*, suggesting an epigenetic control on the expression levels of *ALDH2* (Fig. 4g). To confirm these data, we assessed by RT-qPCR the expression of both *BRIP1* and *ALDH2* in 9 breast cancer cell lines representing different breast tumor subgroups [66] (Supplementary Fig. 9a). In 7 out of 9 cell lines, we measured low to undetectable levels of *ALDH2* mRNA, along with increased *BRIP1* expression, recapitulating the inverse pattern of expression highlighted by PARIS. Consistent with the gene expression analysis, we observed high ALDH2 protein levels in SK-BR-3 and MDA-MB-468 and almost undetectable levels in MCF-7, HCC1954, and HCC1937 (Fig. 5a). Conversely, cell lines with low ALDH2 protein levels show increased BRIP1 protein as compared to SK-BR-3 and MDA-MB-468 (Fig. 5a).

To investigate if the predicted dependency on *BRIP1* in the absence of *ALDH2* expression represents a potential tumor vulnerability, we divided breast cancer cell lines into two groups: i) defined as the “control group”, comprises SK-BR-3 and MDA-MB-468, which express high levels of *ALDH2*; ii) defined as the “dependency group”, comprises MCF-7, HCC1954, and HCC1937, which show low levels of *ALDH2* (and an inverse pattern of *BRIP1* expression). Next, we tested the capability of our model cell lines to form colonies upon transfection of two independent gRNAs targeting *BRIP1,* both of which showed consistent downregulation of BRIP1 protein levels. We did not apply exogenous ACE as additional stress since the predicted data were retrieved from CRISPR-Cas9 screens performed in the absence of drug treatments and endogenously-produced ACE can provide a sufficient stimulus to trigger DNA damage [63, 65]. Consistent with our prediction, we observed a significant reduction (*p* ≤ 0.01, Mann–Whitney U test) in the number of colonies upon *BRIP1* KO in the three cell lines belonging to the dependency group, while mild or no effects were observed in the control group cell lines (Fig. 5b, c, Supplementary Fig. 9b, c). In addition, knockout of both *BRIP1* and *ALDH2* genes in the control cell lines SK-BR-3 and MDA-MB-468 led to a reduction in colony number comparable to the effect observed in the dependency cell lines, whereas no difference was observed when only one of the targets was ablated (Fig. 5d). These data further confirmed that the phenotypic effects observed upon genetic inhibition of *BRIP1* are dependent on *ALDH2* levels.

Both BRIP1 and ALDH2 are involved in protecting cells from ACE-induced DNA damage, resulting in DNA double-strand breaks (DSBs) if not properly resolved. We hypothesized that inhibiting both protective axes could lead to an unsustainable increase in DSB formation, explaining the observed phenotypic effect. Of note, except for SK-BR-3, the tested cancer cell lines are characterized by heterozygous mutations with unknown impact in genes encoding different components of the FA pathway (Supplementary Fig. 9d). Therefore, to directly test the interaction of the *ALDH2- BRIP1* dependency without potential confounding factors due to the cancer cell genetic background, we used the genetically stable RPE-1 ^*TP53*−/−^ cells as a model. We modeled *ALDH2* deficiency by generating an *ALDH2* knock-out cell line (Supplementary Fig. 10a). To assess the effects of *BRIP1* and potentially other components of the FA complex on the fitness of *ALDH2* KO cells in an unbiased manner, we performed a CRISPR-Cas9 screen using a custom designed pooled library that mainly targets DDR-related genes (Fig. 5e). In addition, we tested sensitivity of RPE-1 ^*TP53*−/−^ cells to exogenous ACE by performing these screens in the presence of 1 mM ACE (Fig. 5f). Consistent with the results from the cancer cell lines, targeting *BRIP1* as well as several other components of the FA pathway, such as *FANCD2* and *FANCI*, significantly impaired the fitness of *ALDH2* KO cells as compared to the WT (Fig. 5e). These effects were exacerbated by the ACE treatment especially for *BRIP1* (Fig. 5f). Consistently, and independent of the pooled screens, colony formation assays showed that simultaneous targeting of *ALDH2* and *BRIP1* leads to a mild but significant decrease in the number of colonies (Supplementary Fig. 10b-d). Confluence analysis performed over a period of 72 h confirmed that cell proliferation was reduced in the double KO (Supplementary Fig. 10e). In addition, we tested the impact of single or combined KOs on the sensitivity of RPE-1 ^*TP53*−/−^ cells to exogenous ACE on colony formation. *BRIP1* KO, but not *ALDH2* KO, increased cell sensitivity to ACE and this effect was mildly elevated by the double KO (Supplementary Fig. 10f). These results indicate that BRIP1 is a major determinant of ACE sensitivity, consistent with the observation that the FA pathway mainly counteracts the genotoxic effects of ACE in mature, differentiated cells, while ALDH2 is dispensable [63].

Next, we explored the DSB response in RPE-1^*TP53*−/−^ cells upon single or combined KO. As proxy, we measured the nuclear foci formation of the histone variant H2AX phosphorylated at Ser139 (γ-H2AX), a well-established marker of DNA damage. Consistent with our hypothesis, we detected increased nuclear foci count upon combined *BRIP1* and *ALDH2* KOs, compared to the single ones (Fig. 5g, h). In parallel, we tested how the cells respond to the KO of *FANCD2*, a component of the FA pathway whose function is reciprocally regulated by BRIP1 [67]. Similar to the effect observed upon *BRIP1*-*ALDH2* KO, the combined KO of *FANCD2* and *ALDH2* led to an increased formation of γ-H2AX nuclear foci (Fig. 5g, h, Additional file 4). Consistent with the increased DNA damage measured upon the double KOs, the number of cells significantly decreased under the same conditions (Supplementary Fig. 10 g). In agreement with the decrease in colony forming capacity upon exogenous ACE treatment in *BRIP1* KO cells, we detected an increase in the number of DNA damage foci under the same conditions (Supplementary Fig. 10 h, Additional file 4). In summary, our data demonstrate that *BRIP1* deletion in low-expressing *ALDH2* cells triggers a dependency, presumably through DNA damage response induced by endogenous ACE.

In summary, using machine learning PARIS predicts cancer dependencies in an unbiased and scalable manner. We exemplified the power of this approach by studying the vulnerabilities of DDR-related genes and identified and validated two previously uncharacterized SL interactions. Our approach extends the catalog of cancer vulnerabilities, and provides a simple, rapid and robust way of testing additional SL interactions.

## Discussion

The increasing availability of multiple datasets combining the genetic makeup of the cancer cell lines with large-scale perturbance screens presents new opportunities for uncovering cancer vulnerabilities. In recent years, several studies presented ways to integrate these data and predict potential SL interactions computationally [7–13]. In this work, we address synthetic lethality prediction as a feature selection problem. The RF algorithm, the core of PARIS, can capture non-linear relationships and provides a robust method suitable for datasets in which the number of features is higher than the number of observations. Importantly, the use of the Boruta algorithm provides a way to overcome the multi-comparison error and the multicollinearity and overall, reduces the effect of randomness. The presence of highly correlated features is common in high throughput gene expression data. For this reason, it can be challenging to disentangle the contributions of collinear/co-expressed genes with respect to the outcome, in this case the dependency scores obtained from the gene knockouts. In multiple regression models this could be translated into a reduction of significance of coefficients. Often, a subset of those “redundant” features is selected to maximize and/or reduce the complexity of the final model. Boruta, being an all-relevant RF feature selection method, in the presence of collinear features, all well-related with the outcome, selects all of them and assigns them importance scores. This prevents removal of potentially important predictors and generates rank-based scores, therefore presents an overall better understanding of the contribution of each feature in explaining the outcome.

We provided a comparison of two types of importance score methods: the Gini and the permutation raw. Although the permutation raw method has a much higher computational cost, it generated the most unbiased and stable results with all the tested datasets. In addition, we showed how the use of a traditional RF algorithm with the corrected Gini importance score method can be adequate in terms of computational time and reliability. We suggest, however, to employ the Boruta algorithm with the permutation raw importance score method for final validations.

Here, we first applied PARIS to discover cancer vulnerabilities among known DDR-related genes and then searched for new SL pairs in the entire genome. In addition to providing the most robust SL pairs based on the importance scores, we also built a shiny app that allows the user to easily browse through the precomputed synthetic lethality network and to set custom filters. One advantage of our approach is that the pipeline can be applied to any set of genes and theoretically, to all the genes for which the dependency scores, expression and mutational status are available. However, we note that expression data is a better predictor than mutation data to explain the dependencies from the CRISPR-Cas9 screens as previously reported [55]. Results from expression datasets showed strong consistencies using different RF algorithms and cohorts. Contrary, mutation data led to more variable results although known SL interactions were correctly selected with high scores. It is likely that mutation-based data suffer from the weak predictability of their functional impact.

Searching for SL interactions among known DDR and related genes, we identified a previously uncharacterized vulnerability of *CDKN2A*-deficient cells to *TYMS* depletion. A possible direct role of CDKN2A p16 on nucleotide synthesis regulation has been proposed [68]. Particularly, p16 knockdown was shown to activate mTORC1 and to increase nucleotide synthesis in an RB-independent manner. TYMS plays an essential role in the de novo thymidine nucleotide synthesis [69]. Because of its indispensable role, it is commonly targeted in combination with platinum-based drugs for the treatment of various cancers such as non-small cell lung cancer and mesothelioma [70, 71]. The most widely used agent, 5-fluorouracil (5-FU) has been used in the clinic for decades, however, concerns regarding its lack of sensitivity to TYMS led to the design of anti-folates such as RTX or PMX [39]. In this work, we used cancer cell lines from lung, breast, thyroid, and head and neck cancers that carry nonsense mutations or *CDKN2A* deletions. Depending on the tissue type and expression levels of *CDKN2A*, *TYMS*, and *TYMP*, cells responded to pharmacological TYMS inhibition to different extents. Resistance to PMX can occur through several mechanisms such as *TYMS* expression levels, or multidrug resistance genes [72–76]. It was reported that *TYMP* overexpression correlates with *TYMS* KO sensitivity in cell lines and antifolate treatment in triple negative breast cancer patients [77]. In addition, TYMP promotes tumor growth and metastasis by preventing apoptosis and inducing angiogenesis [78]. Our results suggest that *CDKN2A* status also contribute to the sensitivity of *TYMS* inhibition. Thus, we suggest that both *TYMP* expression and *CDKN2A* status should be monitored in order to better predict PMX sensitivity. Indeed, using cancer cell lines to confirm the TYMS dependency predicted by PARIS, we discovered that the most robust effects can be observed in those lines where *TYMP* is expressed at high levels and *CDKN2A* is mutated or deleted. We note, however, that these effects can be more complex, and tissue type specific. For instance, based on DepMap data we observed a significant cooperation between *TYMS* and *CDKN2A* in esophageal cancer cell lines. However, in lung tumors neither *TYMP* levels nor *CDKN2A* status was able to explain *TYMS* KO sensitivity. In the future it might be interesting to understand if additional players (e.g. other components of the thymidine salvage pathway) can contribute to *TYMS* sensitivity in these tumors. On the other hand, our results may help to better stratify patients affected by esophageal tumors for which PMX use has been tested in clinical trials however, only showing limited success [79]. With the growing understanding of anti-folate sensitivity, better stratification of patients that can benefit from existing TYMS inhibitors may improve therapeutic outcome.

Analysis of the dependency scores between the DDR gene cohort and the whole genome revealed a dependency on *BRIP1* for cells expressing low *ALDH2* levels. Decreased *ALDH2* expression is commonly found in human tumors (http://gent2.appex.kr/gent2/) and is associated with poor cancer prognosis. Silencing of the *ALDH2* locus by DNA methylation has been reported as a mechanism of *ALDH2* downregulation in lung adenocarcinoma [80–82]. Consistently, we found *ALDH2* promoter is hypermethylated in the panel of low-*ALDH2* expressing breast cancer cell lines used in this study.

Upregulation of FA pathway genes is frequently observed in tumors and found to be associated with chemo-resistance [83]. Previous studies have demonstrated that genetic loss of FA pathway components exacerbates ACE-mediated genotoxicity in mouse hematopoietic stem cells [62, 63, 65]. These studies provided important evidence for understanding the impact of ACE-induced DNA damage and the protective role of ALDH2 in the context of FA, a genetic disease leading to bone marrow failure and developmental disorders. Here we show that loss of *ALDH2* induces a dependency on *BRIP1*. We observed mild but consistent effects in both cancer cell lines and genetically stable epithelial cells. While *BRIP1* represented the highest confidence hit predicted by PARIS, analysis of human tumor samples highlighted a consistent upregulation of FA genes in a low-*ALDH2* expression background. Accordingly, the combined inhibition of *FANCD2* and *ALDH2* in RPE-1 cells allowed us to recapitulate the same effects on decreased colony formation and elevated DNA damage that we observed upon *BRIP1*-*ALDH2* KO. Therefore, our results help strengthen the connection between the function of the FA pathway and the role of ALDH2, also consistent with a recent study in Acute Myeloid Leukemia [84]. Overall, we suggest that in a cancer context, low *ALDH2* expression can be used as a parameter to predict treatment outcome of drugs targeting the FA pathway, which can be potentially developed for clinical use [83–86]. Specifically, we propose that our results represent the basis for future investigations of the role of BRIP1 as a potential cancer therapeutic target in an in vivo setting. Although BRIP1 inhibitors are not currently available, there is increased recognition of the therapeutic role of DNA helicases [87], and a deeper understanding of individual helicases’ structure and property may pave the way for the development of specific drugs. In conclusion, our results shed light on a cancer dependency that may help establishing personalized therapeutic approaches.

## Conclusions

We present a computational pipeline to infer SL interactions and vulnerabilities in cancers based on RF feature selection algorithms. The main advantage of PARIS is the ability to select all the relevant features to explain the observed dependency after a gene knockout and to assign them an importance score. We demonstrated how this score is proportional to the likelihood of observing a strong gene interaction and, ultimately, a synthetic lethality. Beyond the main aim to retrieve “one to one” SL relationships, PARIS, due to its nature, can provide additional hints for cases in which multiple factors contribute to the increased sensitivity to a specific gene (e.g. *TYMS* and *WRN*), thus it can also uncover multifactorial vulnerabilities. While we illustrated PARIS’ capacity to predict dozens of already well-studied SL interactions as well as to uncover previously undescribed ones, some aspects of our approach can be further improved in the future. For instance, a different implementation of the pipeline may reduce the computational runtime or additional perturbation screen data can be integrated for more robust analyses. Importantly, since the PARIS pipeline is easy to implement, updated versions can be continuously built thanks to the continuous and rapid expansion of available omics data and CRISPR/Cas9-based screens. This will become increasingly important to spot additional vulnerabilities in cases where more observations, i.e. cell lines carrying a particular deficiency, are required to obtain meaningful results. Overall, our approach offers an original and reliable solution to inference of vulnerabilities in cancer.

## Methods

### PARIS bioinformatic pipeline

The CRISPR-Cas9 screens dependency scores, expression and mutation data were downloaded from the DepMap consortium website (version DepMap19Q3). Single nucleotide variants pathogenicity was predicted with FATHMM-MKL and only mutations with a coding score higher than 0.7 or annotated as damaging or TCGA hotspots were labeled as pathogenic. The presence of a pathogenic mutated gene for each cell lines was coded like 0 = not mutated and 1 = mutated. RNAseq log2(TPM + 1) gene expression for protein coding genes was used.

In order to select mutated genes and gene expression explaining the gene dependency across all the cell lines, a RF-based feature selection step was performed. Particularly, for each gene in the CRISPR-Cas9 screen the Boruta algorithm was run four times, using alternatively the Gini or the raw permutation importance scores and the mutation or the expression data as independent variables. The maximum number of iterations of the Boruta algorithm was set to 500. As a third feature selection approach, a RF algorithm was used with the corrected impurity (corrected Gini) as importance score.

In each loop Boruta assigns a hit (+ 1) to each feature showing an importance score higher than shadowMax. For each feature, hits are counted until they become either significantly higher or lower than what expected by random attempts (estimated as the cumulative distribution function of a binomial distribution with a probability of success by a chance of 0.5). In the first case, the feature is confirmed and in the second one is rejected and removed. The *p*-values are adjusted with the Bonferroni method and a *p*-value cut-off of 0.01 is used. Non-significant features that are neither confirmed nor rejected, are tested again in the next iteration.

The features selected as important to explain the dependency score were extracted and the Pearson correlation coefficient was calculated for each pair of genes to understand the direction of the relationship. Positive correlations were selected from the mutation-dependency pairs and negative correlation from the expression-dependency one. Self-pairs were also removed.

The importance scores were scaled by group between 0 and 1, where 1 was the higher score in the group.

The pipeline was run on a 64 bit Ubuntu (version 16.04) system, with Intel® Xeon® Gold 6152 CPU @ 2.10 GHz and 1.47 TB memory. For the DDR vs DDR cohort, the computational time for each gene model was ~ 2 min with the permutation raw as importance score method and ~ 15 s with the Gini one. For the DDR vs ALL cohort, each gene model required ~ 15 min with the permutation raw and ~ 3 min with the Gini method. The total time needed to complete the entire DDR vs ALL cohort (4 models for each gene; expression/mutation and two importance score methods) was ~ 13 days.

Except for FATHMM-MKL, all the steps were performed in R. Boruta (version 7.0.0) and ranger (version 0.12.1) packages were used for the RF feature selection step.

### Analysis of gene pairs

The scaled importance scores of the selected gene pairs were plotted as a density distribution and as a histogram grouped by scoring methods (Gini, permutation raw and corrected Gini) and features cohorts (expression or mutation). To set a threshold of confidence, we proceeded as follow. The head/tail breaks algorithm is specifically applied to cluster long tailed distributions. Briefly, the data values, the scaled importance scores in this case, are divided into greater (head) or less (tail) than the mean and the first subset is used as next distribution and the new mean is computed. The process is recursively applied until the head is not a minority of the distribution anymore (length (head)/length (tail) <  = 40%). This last break point is used as a threshold to identify high-confidence pairs.

In order to compare the different importance scores methods only the gene pairs selected by all the three approaches were used in the next analysis.

Each selected gene pair was labeled as “high-confidence” if its scaled importance score was above the threshold for any of the methods and as “low-confidence” if its scaled importance score was below the threshold for all of the methods.

For each selected gene pair, the combined score from STRING database was retrieved if the interaction between the two genes was available. The frequency of interacting genes over the total selected in the different groups was also calculated.

Potential SL interactions were prioritized as follows: gene pairs above the mentioned cutoff were ranked based on their scaled raw permutation importance score. Additionally, genes with a coefficient of variation of the dependency score distribution lower than 0.3 were considered essential and filtered out.

### R shiny app

The selected gene pairs out from the feature selection step were saved into a.csv file reporting: the two gene names, the Pearson correlation coefficient, the importance score from the RF feature selection, the importance score methods and the features source as group and the scaled importance score. A.csv file containing the gene name, the median, coefficient of variation, standard deviation and range of the dependency scores of all screened genes was also generated.

An R shiny app was built to allow easy visualization. The data were represented as an interactive directed graph in which each node represent a gene and each edge a relationship; the arrow starts from the mutated or dysregulated gene and arrives to the dependent gene. The color of the edge indicates the source of the evidence (mutation or expression) whereas the width is proportional to the absolute value of the Pearson correlation coefficient. The color of the node shows the median of the dependency score of the gene in a grey scale to easily identify essential genes.

The shiny app allows users to filter the gene pairs selecting one or both the two feature cohorts (mutation and/or expression) and applying independent thresholds for them, selecting one of the importance score methods (Gini, permutation raw, corrected Gini) and applying the filtering step based on the Pearson correlation coefficient (only negative correlation for expression and only positive correlation for mutation). The network is redrawn in real-time accordingly to the selected filters and can be exported as a.png file. Cytoscape-compatible network and nodes.csv files can be also downloaded.

The filtering step based on the direction of the relationship can be also bypassed to investigate all the possibilities, e.g. to investigate the dependencies in the case of oncogenic expression.

The R shiny app was developed in R using the shiny (version 1.5.0) and visNetwork (version 2.0.9) packages.

### TCGA and GTEX data analysis

TCGA and GTEX *BRIP1* and *ALDH2* expression data were retrieved from the Xena browser (https://xenabrowser.net/). Correlation was calculated using the Pearson coefficient.

### TCGA Differential gene expression analysis

TCGA data were retrieved using the TCGAbiolinks R package (version 2.14.1). Samples were ranked based on their *ALDH2* expression level. The top and bottom 2% of the samples were used as high and low expressing *ALDH2* groups. The differential gene expression analysis was performed with DESeq2 (version 1.26.0). Genes with an adjusted pvalue < 0.01 were considered to be differentially expressed. EnrichR (version 2.1) was used for the enrichment analysis.

### Cell lines and cell culture

Cell lines were maintained in a humified incubator at 37 °C, 5% CO_2._ A table of the cell lines used in this study and their culturing media is listed in Supplementary Table 1.

### Generation of stable cell lines

#### Cas9-positive cell lines

A table of cell lines and sources used in this study is listed in Supplementary Table 1 or detailed in [88]. Briefly, cells were infected using hEF1α-TurboGFP-Cas9 Nuclease viral particles (Dharmacon VCAS11864), according to manufacturer’s specifications. GFP-positive cells were sorted using FACS Aria II instrument (BD Biosciences). Alternatively, cells were infected using Inducible-hEF1α-Blast-Cas9 Nuclease viral particles (Dharmacon VCAS11864), according to manufacturer’s specifications and selected with blasticidin.

#### TYMP-positive cell lines

Cells were infected with lentiviral particles expressing TYMP-GFP (Twist Biosciences – Supplementary Data). GFP-positive cells were sorted using FACS Aria II instrument (BD Biosciences).

#### ALDH2 and CDKN2A KO

RPE-1^*TP53*−/−^ cells stably expressing an inducible Cas9 transgene were transfected with a gRNA targeting ALDH2 or CDKN2A (sequence provided in the Supplementary Table 2), in the presence of 1 μg/ml doxycycline. Three days post transfection cells were seeded on 96-well plates for single clone formation. The KO efficiency of selected single cell-derived clones was assessed by Western blot. Two validated KO clones were mixed and used for downstream experiments.

#### C-MYC-positive cell lines

RPE-1^*TP53*−/−^ or RPE-1^*TP53*−/− *CDKN2A*−/−^ Cells were infected with lentiviral particles expressing cMYC-BFP (Twist Biosciences – Supplementary Data). BFP-positive cells were sorted using FACS Aria II instrument (BD Biosciences).

### Lentivirus generation

Lentiviral plasmids pMD2G (12,259) and psPAX2 (12,260) were obtained from Addgene. A lentiviral plasmid expressing *TYMP* cDNA was purchased from Twist Biosciences. One μg of each plasmid was transfected to 293FT cells using Lipofectamine 3000 according to manufacturers’ protocol. Viral particles were collected 72 h post transfection, filtered using a 0.45 μm low protein binding membrane Steriflip HV/PVDF (Millipore) and stored at –80 °C.

### Transfection of gRNAs

Exponentially proliferating cells at 70–80% confluency were transfected with 2.5 pmol gRNA complexes and 0.3 μl of Lipofectamine RNAiMAX/96 well (Invitrogen 13,778,150) according to the manufacturer’s protocols. A detailed list of gRNAs is provided in the Supplementary Table 2.

### Pooled CRISPR screens

Custom designed lentiviral library was generated by Cellecta. Lentiviral particles were produced by co-transfecting 293FT cells with 50 μg of custom designed library (Cellecta), 50 μg of pMD2.G (Addgene, 12,259), and 75 μg of psPAX2 (Addgene, 12,260) using Lipofectamine 3000 (Thermo Fisher Scientific, L3000001) following manufacturer’s specifications. After 60 h, viral supernatant was collected and filtered through a 0.45 μm low protein binding membrane Steriflip HV/PVDF (Millipore).

Ten million RPE^*TP53*−/−^ and RPE^*TP53*−/−,ALDH2−/−^ cells per library and per replicate, aliquoted to 600.000 cells per well of a 6-well plate, were spinfected with the lentiviral library at 2,000 rpm, for 2 h at 37 °C, with the addition of 8 μg/mL polybrene (Sigma-Aldrich, TR-1003) to reach a multiplicity of infection of 0.3. Cells were left to recover in fresh medium for 24 h incubated at 37 °C, 5% CO_2_. The infected cells were selected with 10 μg/ml puromycin (Thermo Fisher Scientific, A1113803) for 3 days. Following puromycin selection, Cas9 was induced with 1 μg/ml Doxycycline (Day0). Cells were incubated in a humidified atmosphere to proliferate for a total of 21 days and subdivided to 2 million cells every three days to maintain appropriate coverage. ACE treatment was started after 6 days of Doxycycline induction by addition of 1 mM ACE and kept throughout the experiment by replenishing the ACE every time the cells were subdivided.

For preparation of the sequencing libraries, genomic DNA was isolated from the initial and final time points using QIAamp DNA Blood Kit (Qiagen, 51,106) following manufacturer’s protocol. Eight μg DNA was used as a template in two separate reactions of 50ul for 12 cycles using plasmid-specific primers for the amplification of the sgRNA coding sequences. An additional round of PCR amplification was run for 10 cycles for the addition of Illumina adaptors and sample specific barcodes. Q5 NEBNext Hot Start polymerase master mix (M0543) was used for the PCR reactions according to manufacturer’s specifications. The primers used for the reactions are depicted in the Supplementary Table 2. PCR reaction products of 390 bps were purified using 0.8X Agencourt AMPure XP (Beckman Coulter, A63880) and eluted in low EDTA TE buffer (Thermo Fisher, 12,090,015). All samples were pooled and sequenced using Illumina NextSeq platform (Illumina), using 75 base single reads.

The cell fitness is calculated based on the gRNA counts at the beginning and at the end of the experiment since the selection process may enrich or deplete sgRNAs from the cell population. Accordingly, the gRNAs that result in loss of cell fitness are expected to be depleted from the cell population at the final time point as compared to the first time point. To count the number of gRNAs in each library, and to perform downstream analyses we used MEMcrispR [89] that enables efficient analysis of genome-wide count-based screens based on linear mixed-effects regression on the initial and final timepoints. For each sample, the sgRNAs were aligned and the number of gRNAs were normalized according to sequencing depth. Afterwards, comparing the Day 6 and Day 21 of each sample, the *P*-values and beta scores that measure the degree of the perturbation effect were calculated for each gene using the mixed effect model. Finally, the beta scores differences between RPE^*TP53-*/−,ALDH2−/−^ and RPE^*TP53*−/−^ were calculated for each gene. Genes with a positive or a negative beta score differences enhance or decrease the RPE^*TP53*−/−,ALDH2−/−^growth compared to RPE^*TP53*−/−^ after being knocked out, respectively. The RankView function from the MAGeCKFlute R package was used to plot the results.

### Cell viability assays

CellTiter-Glo (Promega) was used to determine cell viability, according to the manufacturer’s protocol. Cells were seeded on a 96-well white plate with a clear flat bottom (Costart®Assay plate, Corning). The GloMax-Multi detection system (Promega) was used as a luminometer to quantify the presence of ATP as an indicator of metabolically active cells.

For thymidine rescue experiments, CellTiter-Fluor (Promega) was used according to manufacturer’s protocol in parallel to CellTiter-Glo experiments as described above.

IC50 values were calculated with a non-linear regression model using GraphPad PRISM.

### Colony formation assay

Three days post gRNA transfection, equal number of cells were plated in 6-well plates. After 12–15 days colonies were stained with 0,2% crystal violet (Sigma-Aldrich V5265) and scanned with a GelCount Scanner (Oxford Optronix). The colony number was quantified using the ImageJ software either by manual counting with a multi-point tool or using a colony area plugin [90].

### RNA extraction, cDNA synthesis and RT-qPCR

RNA was isolated using RNeasy Plus Mini kit (Qiagen), according to manufacturer’s instructions. cDNA synthesis was performed using the qScript cDNA Synthesis Kit (VWR International). RT-qPCR was carried out using QuantStudio 3 Real-Time PCR System, following manufacturer’s instructions. The oligonucleotide list is presented in Reagents and Tools Table. 36B4 was used as housekeeping gene for internal normalization.

### Western blot

Cells were collected in ice-cold PBS. Cell pellets were lysed in RIPA buffer (Cell Signaling, 9806S) supplemented with 0,2% SDS, PMSF and protease/phosphatase inhibitor cocktail (Cell Signaling, 9806S). Twenty μg of whole cell lysates was resolved on NuPAGE 4–12% Bis–Tris Protein Gels (Invitrogen) and transferred onto PVDF membranes (neolab Migge GmbH, IPFL00010). Membranes were blocked in 5% BSA and incubated overnight with primary antibodies (Reagents and Tools Table). Anti-mouse or anti-Rabbit IgG, HRP-linked Antibody (Cell signaling, 7076; 7074) were used as secondary antibodies and signals were detected using Vilber FUSION FX7 (Vilber Lourmat). VINCULIN and β-ACTIN served as loading controls.

### Immunofluorescence

Cells were seeded in 96 well glass bottom plates (Cellvis, P96-1.5H-N), fixed for 10 min with 4% PFA at room temperature, washed 3 times with D-PBS, permeabilized for 10 min with 0,25% Triton-X-100 in D-PBS and blocked with 10% FBS—0.1% Triton-X100 for 1 h at room temperature. Cells were then stained overnight at 4 degrees with γ-H2AX primary antibody (Merck Millipore 05–636) diluted 1:500 in 3% BSA/D-PBS, washed 3 times with D-PBS and stained with secondary antibody (Alexa Fluor-594, goat anti mouse, 1:1000) and Hoechst (1:4000) in 3% BSA/D-PBS for 1 h. Cells were stored in the dark at 4 °C until observed under the microscope.

### Microscopy

Fluorescent images were acquired on a Nikon Ti-E automated epifluorescent microscope. The microscope was equipped with a DS-Qi2 camera and a Sola nIR LED lamp. The filter sets were provided by Nikon for DAPI (DAPI-A-2360A), Alexa Fluor 568 (Cy3-A-4040C)) and Alexa Fluor 647 (LED-Cy5-5070A). The objectives used were P-Fluor 40X, numerical aperture 0.60 (Nikon). We used a hardware base autofocusing system called the perfect focus system (PFS) from Nikon to automatically focus the cells on the field of view. For each replicate, we imaged 2 wells per condition with multiple images (a minimum of 4 and a maximum of 9) per well.

For quantification of γ-H2AX foci, and CyclinA levels, images were analyzed by ImageJ with custom made macros. Briefly, the nuclei were segmented from DAPI channel image by automated thresholding and watershed procedure to split touching nuclei. For analysis of CyclinA levels, the mean intensities of the nuclear CyclinA were measured based on the segmented nuclei in each image.

For counting γ-H2AX foci, similar nuclear segmentation steps were applied. Based on the segmented nuclei in each image then we applied another round of thresholding and segmentation for γ-H2AX channel. Downstream analyses of the foci counts were performed in R.

### Live cell imaging

Three days post gRNA transfection, equal number of cells were plated in 96-well plates. Cell proliferation was monitored over time using the Incucyte S3 scanner. Phase contrast images were acquired every 12 h using a 10 × objective and analyzed using the Incucyte S3 Live-Cell Analysis System.

### Statistical analyses

The applied statistical tests are specified in each figure legend.

## Supplementary Information

**Additional file 1.**

**Additional file 2.**

**Additional file 3.**

**Additional file 4.**

**Additional file 5.**

## Data Availability

All data associated with this study are presented in the paper and DepMap data were downloaded from https://depmap.org/ (version DepMap19Q3). The R shiny apps showing the preprocessed PARIS results can be downloaded at https://github.com/sbenfatto/PARIS_DDR_vs_DDR and at https://github.com/sbenfatto/PARIS_DDR_vs_ALL and run on a local computer. They are also freely usable at https://sbenfatto.shinyapps.io/paris_ddr_vs_ddr/ and https://sbenfatto.shinyapps.io/paris_ddr_vs_all/. Source data underlying Fig. 2 is provided as Additional file 1. Source data underlying Fig. 3d is provided as source Additional file 2. Source data underlying Fig. 4 is provided as Additional file 3. Source data underlying Fig. 5h and Supplementary Figs. 10 g and h are provided as Additional file 4. The PARIS pipeline repository is available at https://github.com/sbenfatto/PARIS, it includes the main functions to run PARIS as well as the ones used to perform the analysis and to generate the plots in the present work. Correspondence and requests for materials should be addressed to B.R.M (mardin@bio.mx).

## Abbreviations

PARIS: Pan-Cancer inferred synthetic lethalities
SL: Synthetic lethal
RF: Random forest
CCLE: Cancer Cell Line Encyclopedia
TCGA: The cancer genome atlas
GTEX: Genotype-tissue expression
PMX: Pemetrexed
dUMP: Deoxyuridine monophosphate
dTMP: Deoxythymidine monophosphate
FA: Fanconi anemia
ICL: Interstrand crosslink
ACE: Acetaldehyde
DSBs: DNA double-strand breaks
RPE-1: Retinal pigment epithelisal
KO: Knock out
