# Supplementary material for: Uncovering cancer vulnerabilities by machine learning prediction of synthetic lethality
Source: Mol Cancer. 2021 Aug 28;20:111. doi: 10.1186/s12943-021-01405-8 (PMC8401190; doi:10.1186/s12943-021-01405-8)
Supplement: Supplementary file 5 — Additional file 5. [file 12943_2021_1405_MOESM5_ESM.docx]

Supplementary Material

# Uncovering cancer vulnerabilities by machine learning prediction of synthetic lethality

Salvatore Benfatto^1#^, Özdemirhan Sercin^1#^, Francesca R. Dejure^1#^, Amir Abdollahi^2^, Frank T. Zenke^3^, Balca R. Mardin^1*^

Supplementary material contains 10 supplementary figures, supplementary figure legends and 2 supplementary tables

# Supplementary Figures and Figure Legends:


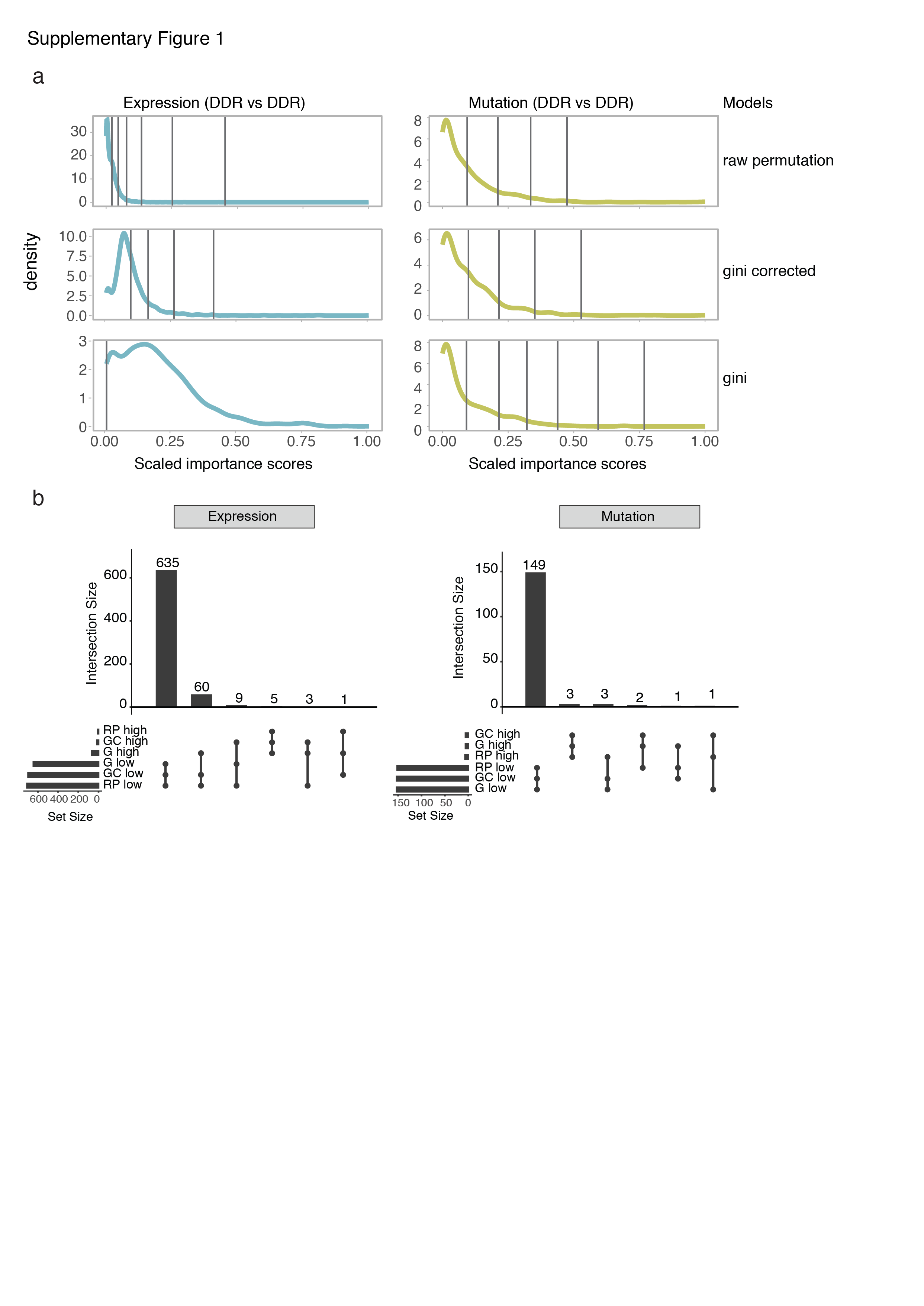


**Supplementary Figure 1 (related to Figure 2)**

**a** Density distribution of the scaled importance scores using expression (blue) and mutation (yellow) data as features and three importance score methods. The vertical lines correspond to the break points of the Head/Tail breaks cluster algorithm by group.

**b** Upset plots showing the intersections of the common selected gene pairs by the three importance score methods divided into high/low confidence (see Methods) for expression (left panel) and mutation (right panel) cohorts.


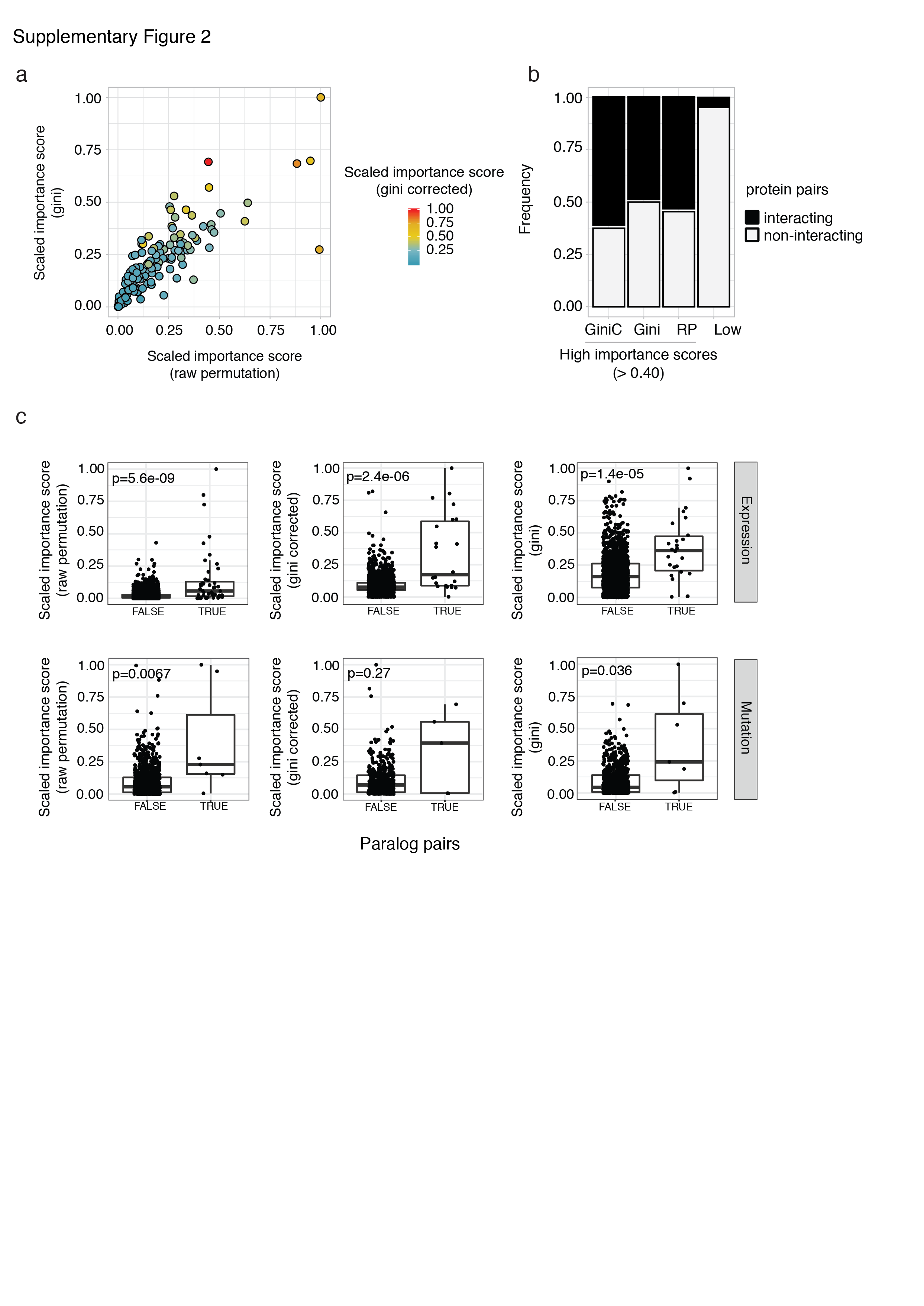


**Supplementary Figure 2 (related to Figure 2)**

**a** Scatterplot showing the Gini – raw permutation correlation on commonly selected gene pairs using mutation features. Dots are colored by the Gini corrected scores.

**b** Percentage of interacting gene pairs over the selected ones in the four groups: high confidence (scaled importance score > 0.4) by the three approaches and with low confidence (scaled importance score < 0.4) using mutation data.

**c** Scaled importance score distributions of selected gene pairs divided based on paralog gene pairs in the two cohorts (expression and mutation). P values are calculated based on Mann-Whitney U test.


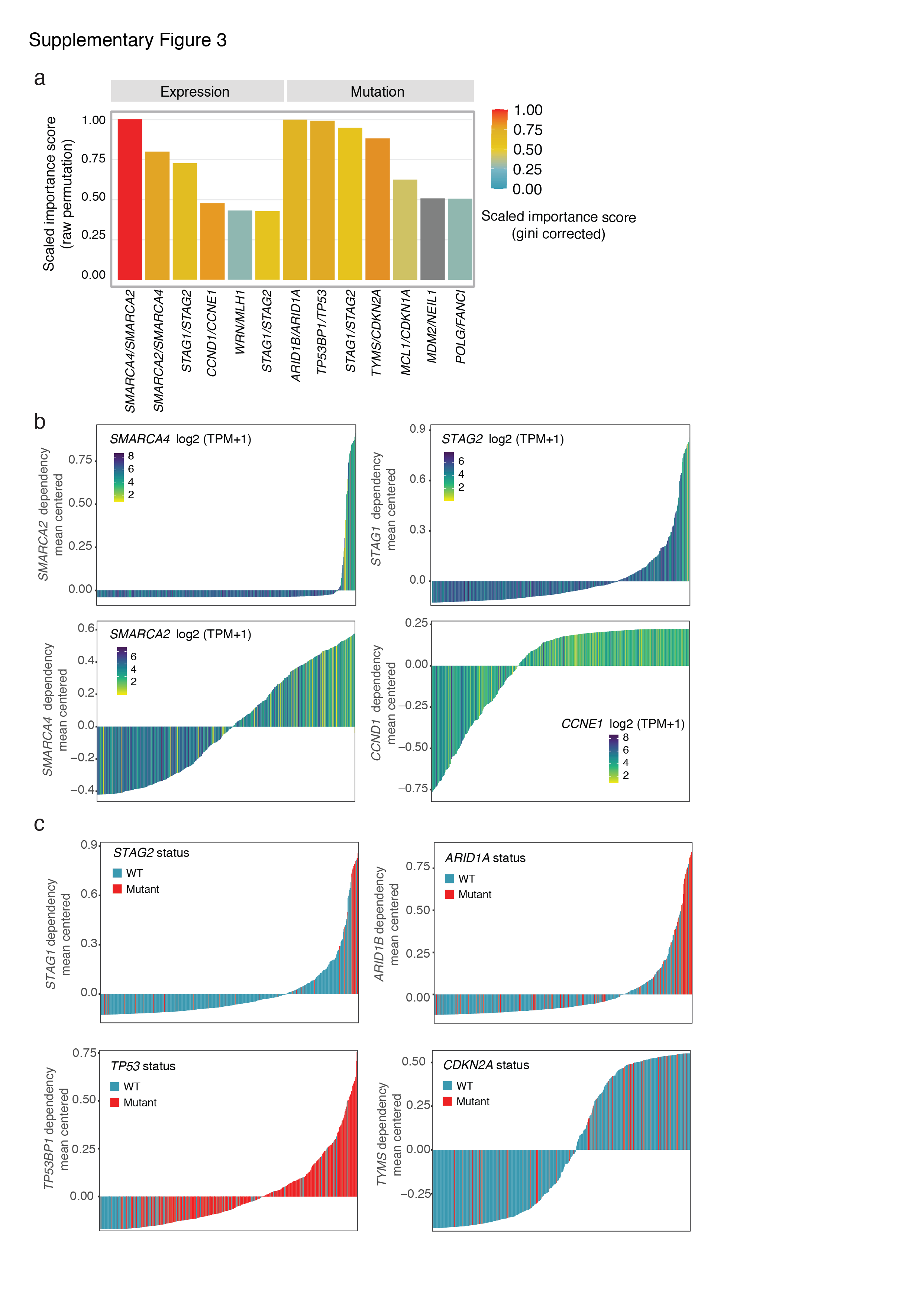


**Supplementary Figure 3 (related to Figure 2)**

**a** Top candidates for synthetic lethality in expression and mutation cohorts. The rank is based on raw permutation scaled importance scores > 0.4 (expression) and > 0.5 (mutation). The Gini corrected values are also showed as color gradient. Bar plots showing examples of high-confidence predicted SL pairs among DDR genes using expression (b) and mutation (c) features. The ranked bars show the dependency scores (mean centered) of one gene across the cancer cell lines and the color shows the expression level (based on RNA-Seq) of the second gene or the mutation status.

**
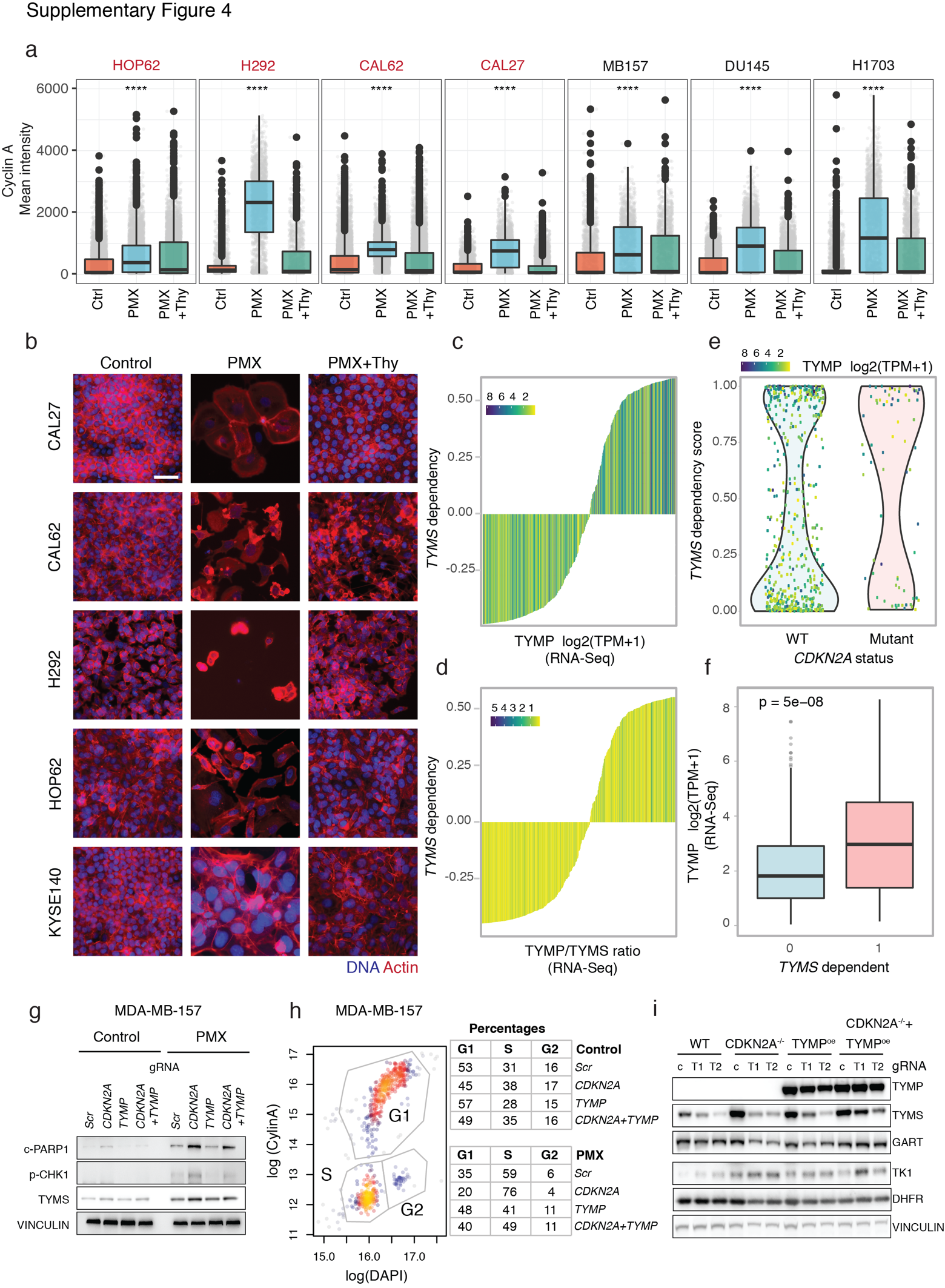
**

**Supplementary Figure 4 (related to Figure 3)**

**a** Boxplot showing CyclinA immunofluorescence staining mean intensity for cell lines used in Fig. 3d. Cells were treated with control (PBS); PMX (5 μM); or 5 μM PMX and 50 μM Thymidine (Thy) for 48h. Images were than processed and analyzed.

**b** Microscopy images of cell lines after 96h of control, 5 μM PMX or 5 μM PMX and 50 μM Thymidine treatment. Scale bar = 20μm.

**c** Barplot showing *TYMS* dependency scores (mean centered) across the cancer cell lines. Bars are ranked and colored by the *TYMP* expression (log2(TPM+1)).

**d** Barplot showing *TYMS* dependency scores (mean centered) across the cancer cell lines. Bars are ranked and colored by the *TYMP*/*TYMS* expression ratio.

**e** Violin plot showing *TYMS* dependency (0 lowest, 1 highest) with respect to mutation status of *CDKN2A*.Color gradient shows the *TYMP* expression level (log2(TPM+1)). Each point represents a cell line.

**f** Boxplot showing *TYMP* expression (log2(TPM+1)) in low and high *TYMS* dependent *CDKN2A* WT cancer cell lines.

**g** MDA-MB-157 cells transfected with the corresponding gRNA as described in (Fig 3g) were treated with PBS or PMX for 96h. Induction of DNA damage checkpoint and apoptosis were tested with phospho-Chk1 (S345) and cleaved Parp1 antibodies, respectively upon PMX treatment.

**h** MDA-MB-157 cells transfected with the corresponding gRNAs as described in (Fig. 3g and Fig. S3g) were treated with PBS or PMX for 48h. CyclinA immunostaining was performed with DNA staining and images were processed and anlysed. Cell cycle profiles were generated using nuclear intensities of CyclinA and DAPI signals. Cells in S, G1 and G2 phases were denoted in the exemplary graph. Percentages were shown in the table.

**i** RPE1 *^TP53-/-; CMYC^* , RPE1 *^TP53-/- ; CMYC;CDKN2A-/-^*, RPE1 *^TP53-/- ; CMYC; TYMP^*, RPE1 *^TP53-/-; CMYC ; TYMP ;CDKN2A-/-^*,cells were transfected with two different gRNA against *TYMS.* 72 hours post transfection*,* western blot was performed for the proteins involved in Thymidine nucleotide metabolism (DHFR, GART, TYMS, TK1 and TYMP). VINCULIN served as loading control.


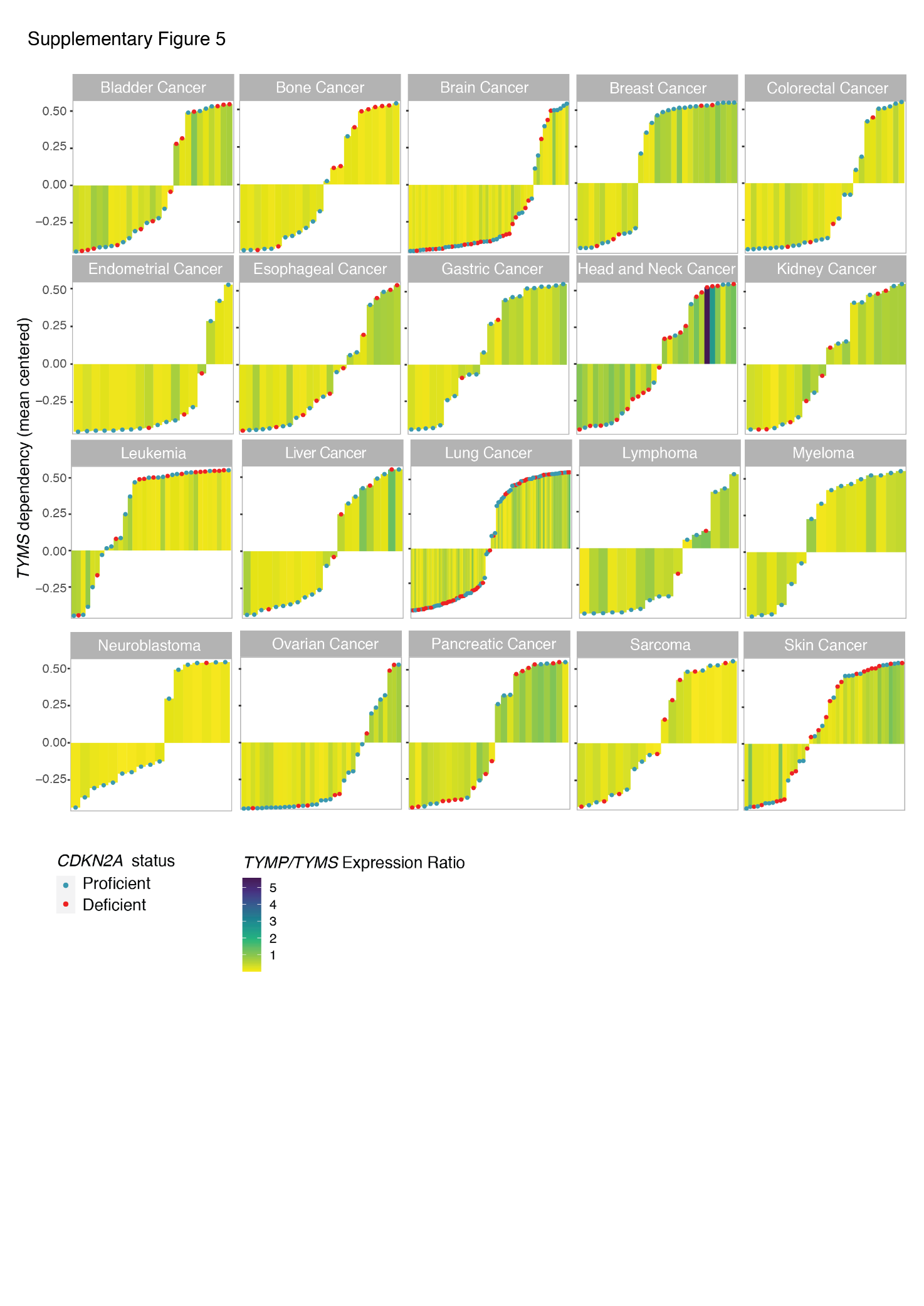


**Supplementary Figure 5 (related to Figure 3)**

Bar plots showing *TYMS* dependency scores (mean centered) across the cancer cell lines grouped by tissue. Bars are ranked and colored by the *TYMP*/*TYMS* expression ratio. Colored dots represent the *CDKN2A* status, denoted with blue (WT) and red (mutant or deleted).


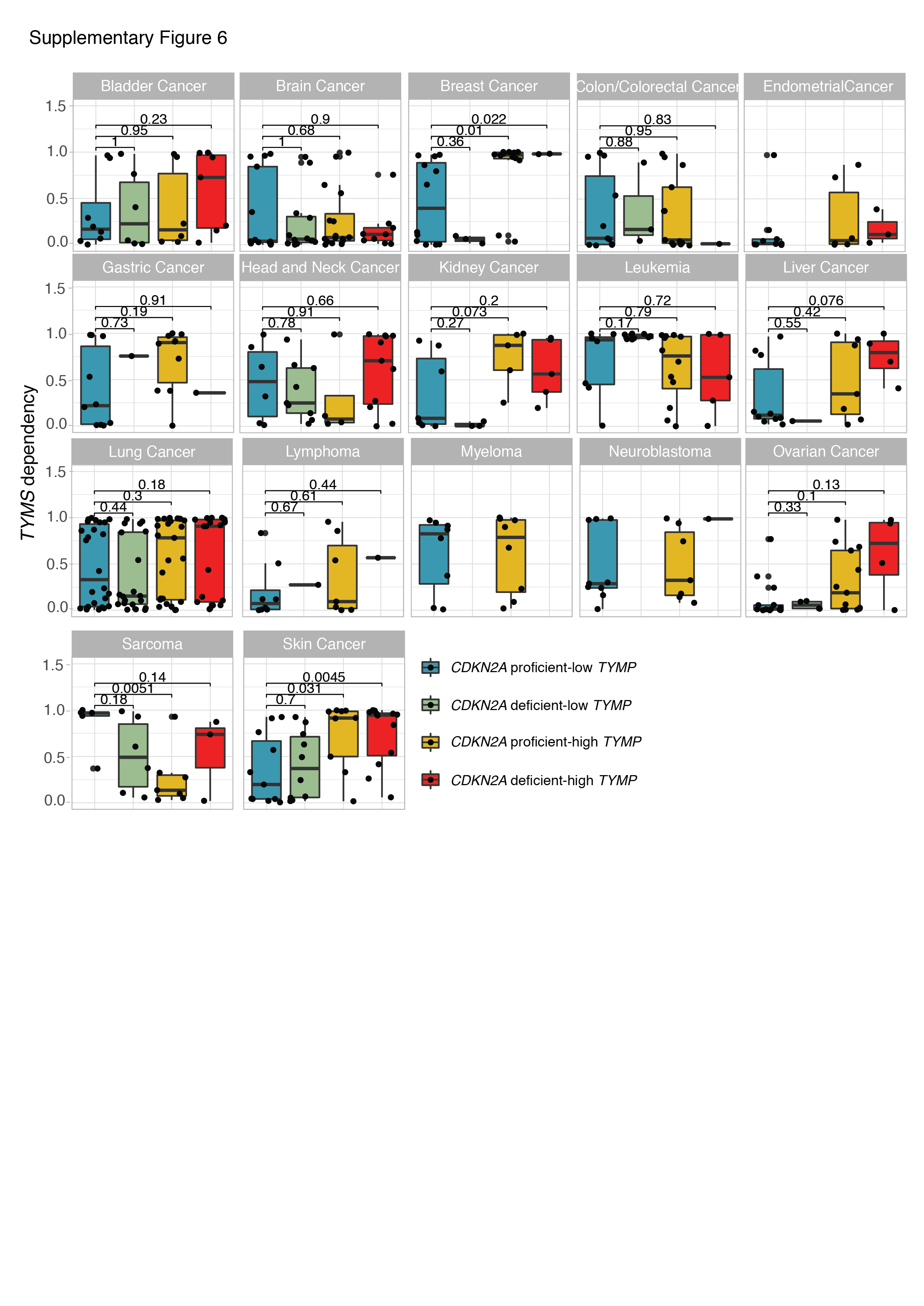


**Supplementary Figure 6 (related to Figure 3)**

Boxplots showing the *TYMS* dependency score distribution by tissue. Cell lines are grouped by their *CDKN2A* and *TYMP* status. *CDKN2A* deficiency/proficiency is defined by the presence of a mutation or copy number loss and *TYMP* status is defined by tissue as high and low expressed using the median (calculated for each tissue separately) as a cut-off. P-values of the Mann-Whitney U test are shown for the compared groups.


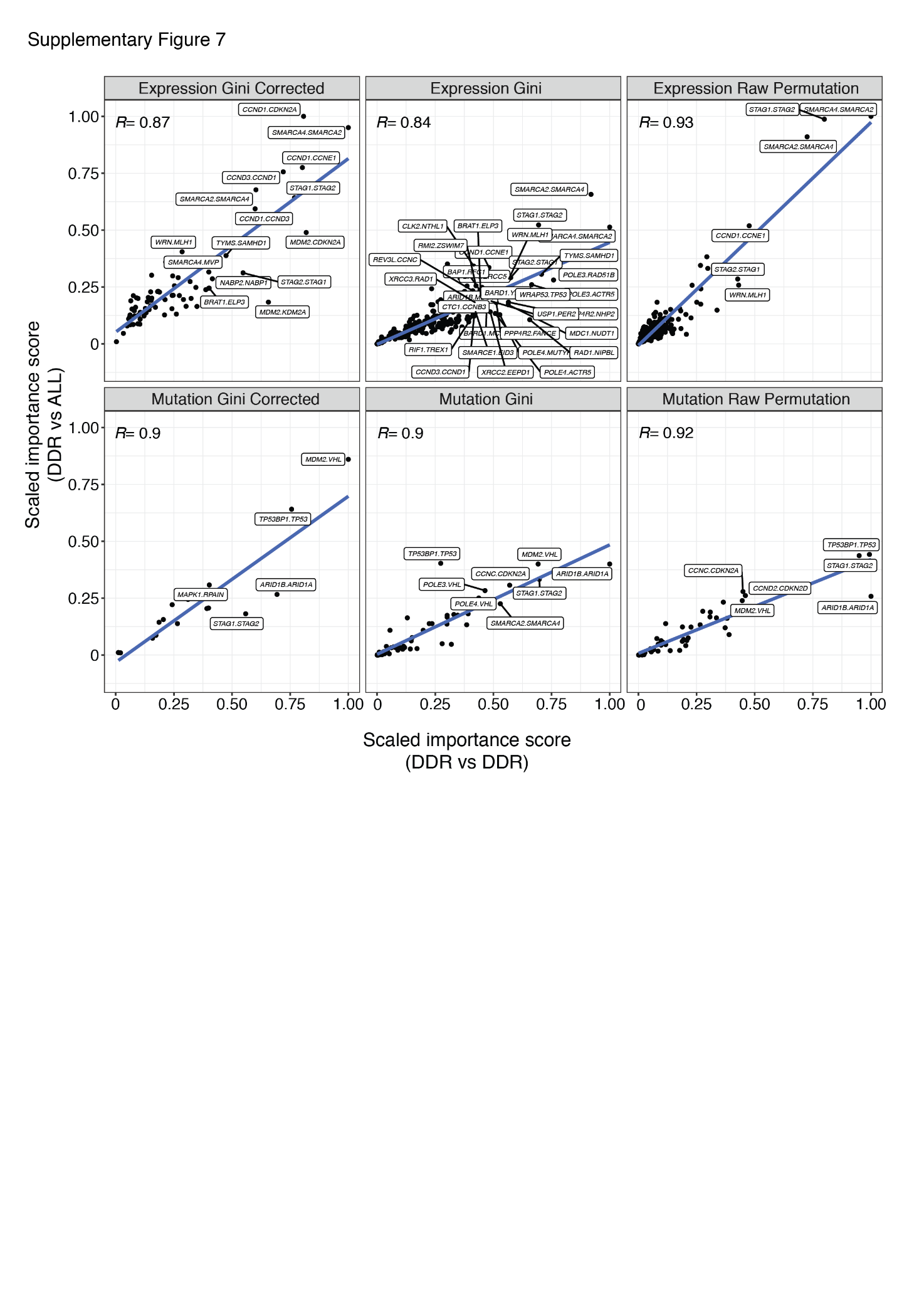


**Supplementary Figure 7 (related to Figure 4)**

DDR vs DDR / DDR vs ALL (entire genome) comparison. The scatterplots show the correlation of the scaled importance scores of commonly selected gene pairs between DDR vs DDR and DDR vs ALL. Plots are grouped by feature type (expression and mutation) and importance score method.


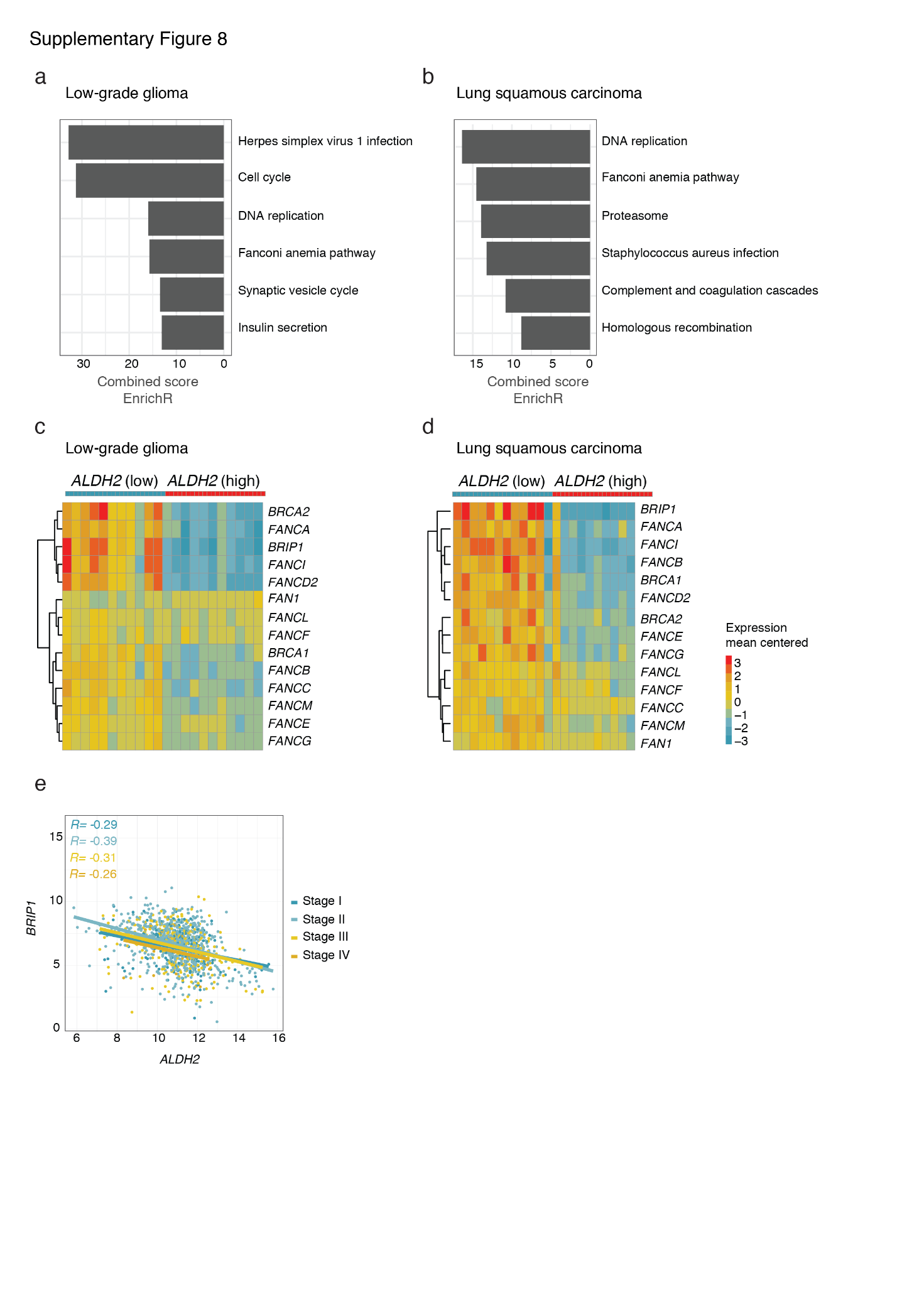


**Supplementary Figure 8 (related to Figure 4)**

Top altered pathways in the enrichment analysis of differentially expressed genes between TCGA samples expressing high or low *ALDH2* in low-grade glioma (**a**) and lung squamous carcinoma (**b**).

Heatmap showing expression levels (mean centered) of the main Fanconi anemia genes of TCGA samples expressing high or low *ALDH2* in low-grade glioma (**c**) and lung squamous carcinoma (**d**).

**e** Scatterplots showing the expression levels of *BRIP1* and *ALDH2* in breast tumor samples separated by tumor stages.


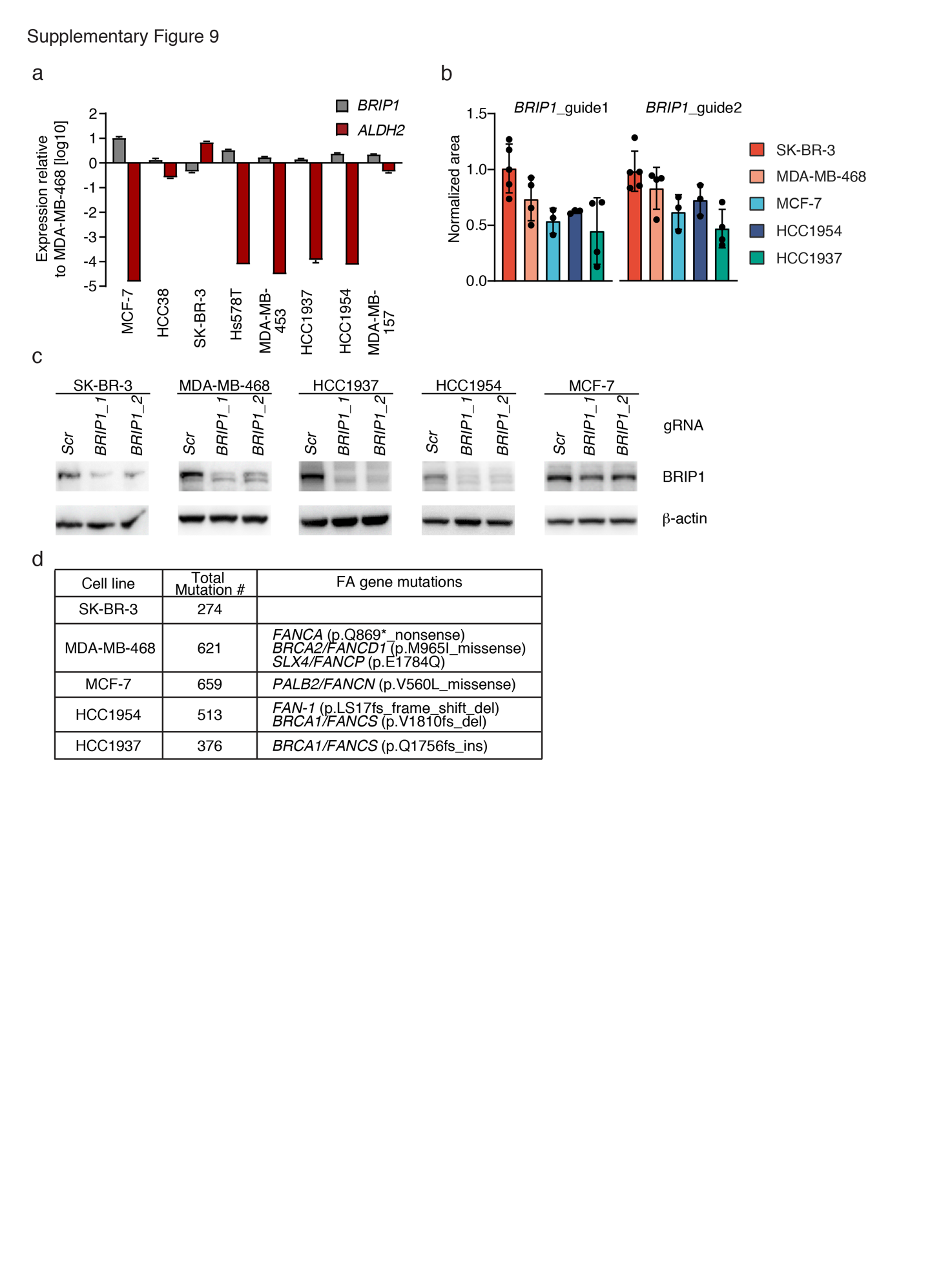


**Supplementary Figure 9 (related to Figure 5)**

**a** RT-qPCR of *BRIP1* and *ALDH2* expression levels in a panel of breast cancer cell lines. Each gene’s expression levels were normalized to those found in the MDA-MB-468 cell line, in which the expression of *ALDH2* and *BRIP1* are comparable. Data are representative of 2 independent experiments. Error bars indicate standard deviation of technical triplicates.

**b** Colony formation assay quantification based on the colony area percentage. The scrambled KO is used for normalization. Each dot indicates independent biological replicates.

**c** Western blot of BRIP1 in the indicated cell lines stably expressing Cas9 and transfected with a scrambled gRNA or 2 independent gRNAs targeting *BRIP1*. β-ACTIN served as a loading control. Cells were harvested 15 days post-transfection, in parallel to the end of the colony formation assays. Images are representative of ≥ 3 independent experiments.

**d** Table indicating the total number of mutations and the mutations in FA pathway genes in the indicated cell lines. Data were retrieved from The Cancer Cell Line Encyclopedia (CCLE).


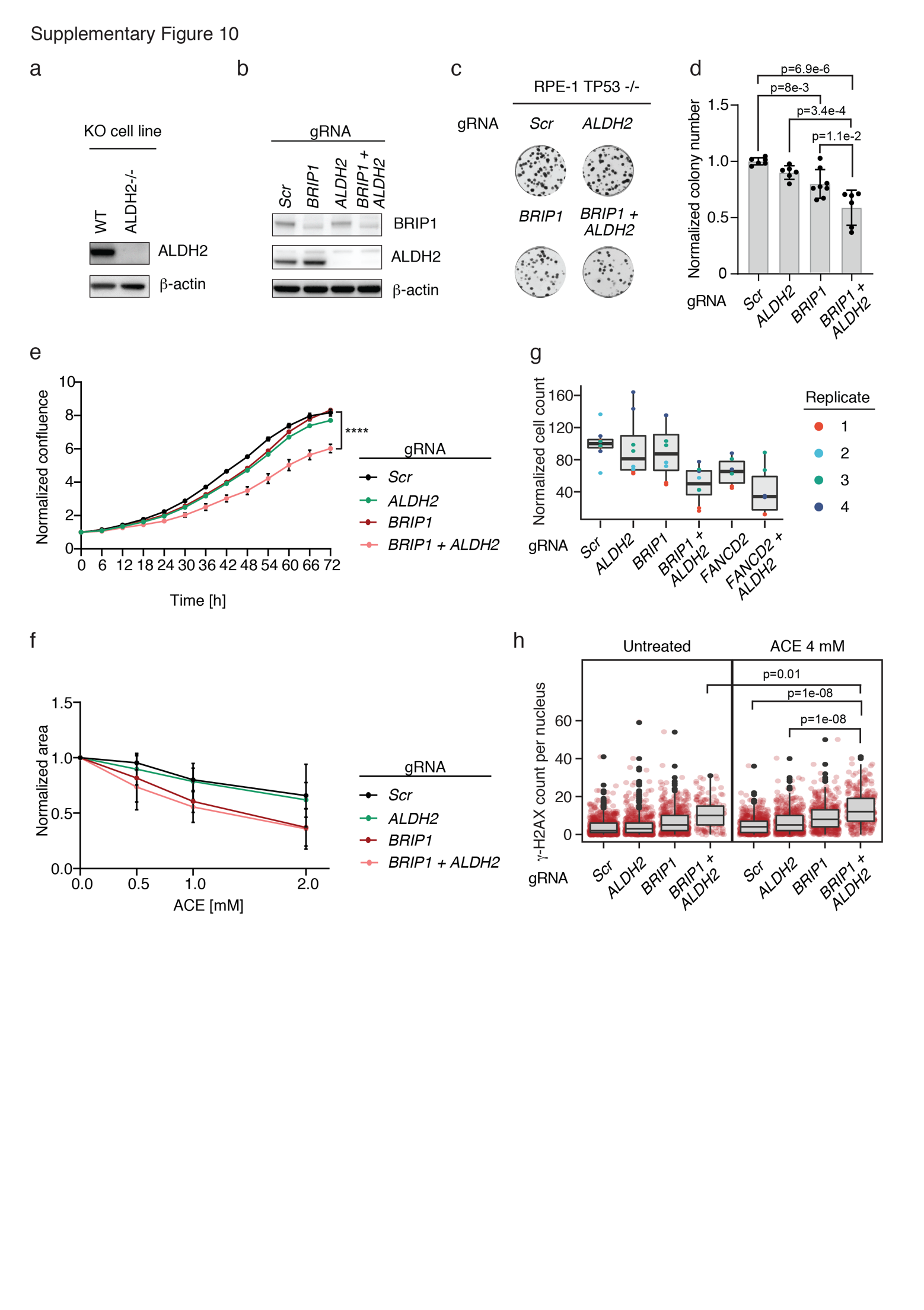


**Supplementary Figure 10 (related to Figure 5)**

**a** Confirmation of *ALDH2* KO in in RPE-1*^TP53-/-^* cells stably expressing Cas9. β-ACTIN served as a loading control.

**b** Immunoblot of BRIP1 and ALDH2 in RPE-1*^TP53-/-^* cells stably expressing Cas9 and transfected with the indicated gRNAs. β-ACTIN served as a loading control. Samples were collected 18 days post-transfection, in parallel to the end of the colony formation assay. Data are representative of 3 independent experiments.

**c** Representative colony formation assay images of RPE-1*^TP53-/-^* cells stably expressing Cas9 and transfected with the indicated gRNAs. Colonies were stained with crystal violet 15-18 days post-transfection.

**d** Colony formation assay quantification based on the colony number. Bars represent mean + standard deviation of 3 biological replicates each performed in technical duplicate. P-values are calculated using a one-way ANOVA test. Significant p-values are indicated.

**e** Cell proliferation analysis in RPE-1*^TP53-/-^* stably expressing Cas9 and transfected with the indicated gRNAs. Live cell imaging was started 3 days post-transfection and performed for 72h. Data are based on cell confluence measurements of 4 technical replicates and are normalized per each condition to the time point 0. P-values are calculated based on a one-way ANOVA test calculated at the 72h time point (**** p ≤ 0.0001).

**f** Acetaldehyde (ACE) titration curve in RPE-1*^TP53-/-^* cells stably expressing Cas9 and transfected with the indicated gRNAs. Data are obtained from the area percentage quantification of colony formation assays performed in the presence of the indicated ACE concentrations. Data are normalized to each KO in the absence of treatment. Each dot indicates the average of 3 independent experiments. Error bars represent standard deviation.

**g** Normalized count of RPE-1*^TP53-/-^* cells based on the nuclei counts. For each experiment, the number of nuclei/well are counted from the DAPI channel and normalized to WT. The count refers to the cells analyzed in Fig. 5h. In the boxplots, centerlines mark the medians, box limits indicate the 25^th^ and 75^th^ percentiles, and whiskers extend to 5^th^ and 95^th^ percentiles. P-values are calculated based on a one-way ANOVA test. The complete list of significant p-values is provided in Source Data File 3.

**h** Quantification of γ-H2AX foci formation in RPE-1*^TP53-/-^* cells stably expressing Cas9 and transfected with the indicated gRNAs. Cell images were acquired 6 days post transfection and where indicated, treated 48h before with 4mM ACE. Each dot represents the number of DNA damage foci/nucleus. Data are representative of two biological replicates. P-values are calculated using a one-way ANOVA test. Selected significant p-values are indicated. The complete p-value list in provided in additional file 4.

**Supplementary Table 1. Cell lines used in this study**

All growth media supplemented with 10% FBS (Gibco 10270-106) and 1% Antibiotic-Antimycotic Solution (Gibco 15240-062)

| Cell Line | Medium | Cas9 Expression | Source |
| --- | --- | --- | --- |
| NCI-H1703 | RPMI 1640  (ATCC modification) (Gibco A10491-01) | hEF1α-TurboGFP-Cas9 Nuclease (VCAS11864)  GFP-positive sorted | ATCC No. CRL-5889 |
| KYSE-70 | RPMI 1640  (ATCC modification) (Gibco A10491-01) | Dox (1μg/ml) inducible Cas9 expression,  Puromycin (8μg/ml) selected | DSMZ No. ACC 363 |
| DU-145 | RPMI 1640  (ATCC modification) (Gibco A10491-01) | Dox (1μg/ml) inducible Cas9 expression,  Puromycin (8μg/ml) selected | ATCC HTB 81 |
| MDA-MB-157 | RPMI 1640  (ATCC modification) (Gibco A10491-01) | hEF1α-TurboGFP-Cas9 Nuclease (VCAS11864)  GFP-positive sorted | ATCC HTB 24 |
| HOP-62 | RPMI 1640  (ATCC modification) (Gibco A10491-01) | Dox (1μg/ml) inducible Cas9 expression,  Puromycin (8μg/ ml) selected | NCI-Frederick Cancer |
| NCI-H292 | RPMI 1640  (ATCC modification) (Gibco A10491-01) | Dox (1μg/ml) inducible Cas9 expression,  Puromycin (8μg/ ml) selected | ATCC No. CRL-1848 |
| HEK293FT | DMEM-GlutaMAX  (Gibco 61965-026) | Dox (1μg/ml) inducible Cas9 expression,  Puromycin (8μg/ ml) selected | Thermo Fisher Scientific |
| CAL-62 | DMEM-GlutaMAX  (Gibco 61965-026) | Dox (1μg/ml) inducible Cas9 expression,  Puromycin (8μg/ ml) selected | Sercin et al 2019 |
| CAL-27 | DMEM-GlutaMAX  (Gibco 61965-026) | Dox (1μg/ml) inducible Cas9 expression,  Puromycin (8μg/ ml) selected | Sercin et al 2019 |
| SK-BR-3 | RPMI 1640  (ATCC modification) (Gibco A10491-01) | hEF1α-TurboGFP-Cas9 Nuclease (VCAS11864)  GFP-positive sorted | ATCC HTB 30 |
| MDA-MB-468 | RPMI 1640  (ATCC modification) (Gibco A10491-01) | hEF1α-TurboGFP-Cas9 Nuclease (VCAS11864)  GFP-positive sorted | ATCC HTB 132 |
| MCF7 | DMEM-GlutaMAX  (Gibco 61965-026) | hEF1α-TurboGFP-Cas9 Nuclease (VCAS11864)  GFP-positive sorted | ATCC HTB 22 |
| HCC1954 | DMEM-GlutaMAX  (Gibco 61965-026) | hEF1α-TurboGFP-Cas9 Nuclease (VCAS11864)  GFP-positive sorted | ATCC CRL-2338 |
| Hs578T | DMEM-GlutaMAX  (Gibco 61965-026) |  | ECACC No. 86082104 |
| MDA-MB-453 | RPMI 1640  (ATCC modification) (Gibco A10491-01) |  | DSMZ ACC 65 |
| M14 | RPMI 1640  (ATCC modification) (Gibco A10491-01) |  | NCI-Frederick Cancer |
| KYSE140 | RPMI 1640  (ATCC modification) (Gibco A10491-01) |  | DSMZ No. ACC 348 |
| KYSE450 | RPMI 1640  (ATCC modification) (Gibco A10491-01) |  | DSMZ No. ACC 387 |
| RPE-1 TP53-/- | DMEM-F12 (1:1) GlutaMax (Gibco 31331-028) | Doxycycline inducible-hEF1α-Blast-Cas9 Nuclease (VCAS11227)  Blasticidine selected | Sercin et al 2019 |

**Supplementary Table 2. Reagents, tools and oligonucleotide sequences used in this study**

| Item | Source | Catalog No |
| --- | --- | --- |
| Anti-ALDH2 | Thermo Fisher | MA5-17029 |
| Anti-BRIP1 | Abcam | ab151509 |
| Anti-CDKN2A (p14) | Cell Signaling | 74560 |
| Anti-CDKN2A (p16) | Origene | TA500036 |
| Anti-Cleaved-PARP (Asp214) | Cell Signaling | 5625 |
| Anti-phospho-CHK1 (Ser345) | Cell Signaling | 2348 |
| Anti-phospho-Histone H2A.X (Ser139) | Merck Millipore | 05-636 |
| Anti-TK1 | Cell Signaling | 8960 |
| Anti-TYMP | Sigma-Aldrich | HPA000530 |
| Anti-GART | Bethyl Laboratories | A304-311A |
| Anti-DHFR | Abcam | ab124814 |
| Anti-TYMS | Cell Signaling | 5449 |
| Anti-VINCULIN | Cell Signaling | 4650 |
| Anti-β-ACTIN | Cell Signaling | 3700 |
| Anti-CyclinA2 | Santa Cruz | sc-271682 |
| Alexa Fluor 568 Phalloidin | Thermo Fisher | A12380 |
| Anti-Mouse Alexa Flour-594 Secondary Antibody | Thermo Fisher | A11005 |
| Anti-Mouse IgG HRP-linked secondary Antibody | Cell Signaling | 7076 |
| Anti-Rabbit IgG HRP-linked secondary Antibody | Cell Signaling | 7074 |
| **Virus Particles** |  |  |
| hEF1α-TurboGFP-Cas9 Nuclease | Dharmacon | VCAS11864 |
| Inducible-hEF1α-Blast-Cas9 Nuclease | Dharmacon | VCAS11227 |
| **Plasmids** |  |  |
| psPAX2 | AddGene | #12260 |
| pMD2.G | AddGene | #12259 |
| TYMP-P2A-GFP | Twist Biosciences |  |
| C-Myc-P2A-TagBFP | Twist Biosciences |  |
| **Primers for CRISPR libraries** |  |  |
| **Name** | **Source** | **Sequence (5’-3’)** |
| Fseq-gRNA  (for sequencing) | IDT | GGCTTTATATATCTTGTGGAAAGGACGAAACACCG |
| P7-RSeqIND  (for sequencing) | IDT | AGATGCACGACGAGACGCAGACGAA |
| FwdU6-1 | IDT | CAAGGCTGTTAGAGAGATAATTGGAA |
| Rev-1 | IDT | CGACAACAACGCAGAAATTTTGAA |
| P5-NFG16 | IDT | AGATACGGCGACCACCGAGATCTACACGAGAT  GGACTATCATATGCTTACCGTAACTTGAA |
| P7-NRG16IND  (N denotes barcodes) | IDT | CAAGCAGAAGACGGCATACGAGATGCACGACGAGACGCAG  ACGAANNNNNNNNAGAGTGTATGTCTGTTGCTATTATGTCTACT |
| **RT-qPCR primers** |  |  |
| **Target** | **Source** | **Sequence (5’-3’)** |
| 36B4_For | IDT | CCCATTCTATCATCAACGGGTACAA |
| 36B4_Rev | IDT | CAGCAAGTGGGAAGGTGTAATCC |
| ALDH2_For | IDT | GAGGTCTTCTGCAACCAGATTT |
| ALDH2_Rev | IDT | AAGGCCTTGTCCCCTTCAG |
| BRIP1_For | IDT | TGCAGGTGTACTATGACGCA |
| BRIP1_Rev | IDT | CACGGGCATTGTCATCTGAG |
| **Synthetic RNA** |  |  |
| Edit-R tracrRNA | IDT | U-002005-05 |
| **crRNA** |  |  |
| **Target** | **Source** | **Sequence (5’-3’)** |
| Scramble | IDT | GACGTCTAGCTGGCTAGCAT |
| BRIP1_1 | IDT | TCACTTACGCCCTCATCTGC |
| BRIP1_2 | IDT | TGTGACGGGTAAGCTTTATA |
| ALDH2 | IDT | GCTGAAGAAGTCTCCGTCAA |
| FANCD2 | IDT | CATCCTCAATGTAAGACTCC |
| TYMP | IDT | GTGGTGGACGTTAAGTTCGG |
| CDKN2A | IDT | GACCCGTGCACGACGCTGCC |
| TYMS_1 | IDT | AAGGACGACCGCACGGGCAC |
| TYMS_2 | IDT | CAAAAAGTCTCGGGATCCAT |
| **Chemicals, kits, and other reagents** |  |  |
| 16% PFA solution | Science Service | 15710 |
| Acetaldehyde | Carl Roth | 3004.1 |
| Albumin Fraktion V, ≥98 % | Carl Roth | 8076.4 |
| Applied Biosystems MicroAmp Optical 96-Well Reaction Plate | Fisher Scientific | 10411785 |
| Benzonase Nuclease | Sigma-Aldrich | E1014-5KU |
| CellTiter-Fluor Cell Viability Assay | Promega | G6080 |
| CellTiter-Glo Cell Viability Assay | Promega | G7573 |
| Crystal violet solution  1%, aqueous solution | Sigma-Aldrich | V5265 |
| DAPI | Sigma-Aldrich | 10236276001 |
| Doxycycline | AppliChem | A2951.0025 |
| Fast SYBR® Green Master Mix | Thermo Fisher | 4385612 |
| Glycin | SERVA Electrophoresis | 23390 |
| Hexadimethrine Bromide | Sigma-Aldrich | H9268 |
| Immobilon-FL PVDF, 0,45 µm | Merck-Millipore | IPFL00010 |
| Lipofectamine RNAiMAX Transfection Reagent | Thermo Fisher | 13778075 |
| Methanol | Carl Roth | 8388.6 |
| Milk powder | Carl Roth | T145.2 |
| NuPAGE 4 to 12%, Bis-Tris, 1.0 mm, Mini Protein Gel, 15-well | Thermo Fisher | NP0323 |
| NuPAGE Antioxidant | Thermo Fisher | NP0005 |
| NuPAGE LDS Sample Buffer (4X) | Thermo Fisher | NP0007 |
| NuPAGE Sample Reducing Agent (10X) | Thermo Fisher | NP0009 |
| Opti-MEM I Reduced Serum Medium | Thermo Fisher | 31985-062 |
| PageRuler, Prestained Protein Ladder, 10 to 180 kDa | Thermo Fisher | 26616 |
| Pemetrexed | Sigma-Aldrich | Y0001539 |
| PMSF  Phenylmethylsulfonyl fluoride | Sigma-Aldrich | 10837091001 |
| Protease/Phosphatase Inhibitor Cocktail | Cell Signaling | 5872S |
| qScript XLT cDNA SuperMix | VWR International | 733-2375 |
| RIPA Buffer | Cell Signaling | CST-9806S |
| RNA and DNase free water | Thermo Fisher | 4387936 |
| RNeasy Plus Mini Kit | QIAGEN | 74134 |
| Genomic DNA Extraction | QIAGEN | 51106 |
| Q5 NEBNext | NEB | M0543 |
| AMPure beads | Beckman Coulter | A63880 |
| TE buffer | Thermo Fisher | 12090015 |
| Sodium dodecyl sulfate solution 20% in H2O | Carl Roth | 1057.1 |
| Supersignal west femto sensitivity substrate | Thermo Fisher | 34095 |
| Thymidine | Sigma-Aldrich | T9250 |
| TRIS base buffer-grade | Carl Roth | AE15.2 |
| Tween-20 | Carl Roth | 9127.1 |
| Whatman gel blotting papers, Grade GB005 | Sigma-Aldrich | WHA10426994 |
| Fetal Bovine Serum | Thermo Fisher | 10270106 |
| Antibiotic/Antimycotic | Thermo Fisher | 15240062 |
| Trypsin-EDTA (0.05%), phenol red | Thermo Fisher | 25300062 |
| **Culture and Immunofluorescence Plates** |  |  |
| 96-well plates with glass-like polymer bottom Black frame | Cellvis | P96-1.5P |
| Corning Costar Assay plate, 96-well white with clear flat bottom | Fisher Scientific | 3903 |
| Corning Costar Flat Bottom Cell Culture Plates, 6 well | Fisher Scientific | 3516 |
| Corning Costar Flat Bottom Cell Culture Plates, 96 well | Fisher Scientific | 3599 |
| Falcon 100 mm TC-treated Cell Culture Dish | neolab Migge GmbH | 353003 |
| **Software** |  |  |
| **Name** | **Source** | **Version** |
| FiJi/ ImageJ | <https://fiji.sc/> | 1.52a |
| FUSION FX software | Vilber Lourmat | VO.07 |
| GraphPad Prism |  | 8.4.3 |
| Incucyte S3 Software |  | V2019B |
| QuantStudio Design & Analysis Software |  | 1.4.3 |
| R | <https://www.r-project.org> | 3.5.1 |
| fathmm-MKL | <https://cran.r-project.org/web/>  packages/drc/index.html | 3.0-1 |
| **Equipment** |  |  |
| Automated Cell Counter | Beckman-Coulter Z2 |  |
| Automated Epifluorescent Microscope | Nikon Ti-E |  |
| Colony Scanner | OxfordOptronix Gelcount |  |
| Evos FL Microscope | Thermo Fisher |  |
| FACSAria II | Becton Dickinson |  |
| FUSION FX7 EDGE | Vilber Lourmat |  |
| Glomax Multi+ | Promega |  |
| Incucyte S3 Live-Cell Analysis System | Essen Biosciences |  |
| Inverted Microscope | Nikon Eclipse Ts2 |  |
| Multidrop Combi | Thermofisher |  |
